# Intracluster Sulphur Dioxide Oxidation by Sodium Chlorite Anions: A Mass Spectrometric Study

**DOI:** 10.3390/molecules26237114

**Published:** 2021-11-24

**Authors:** Chiara Salvitti, Federico Pepi, Anna Troiani, Giulia de Petris

**Affiliations:** Dipartimento di Chimica e Tecnologie del Farmaco, “Sapienza” University of Rome, 00185 Rome, Italy; federico.pepi@uniroma1.it (F.P.); giulia.depetris@uniroma1.it (G.d.P.)

**Keywords:** cluster reactivity, sulphur dioxide, sodium chlorite, mass spectrometry, ion-molecule reactions, oxidation reactions

## Abstract

The reactivity of [NaL·ClO_2_]^−^ cluster anions (L = ClO_x_^−^; x = 0–3) with sulphur dioxide has been investigated in the gas phase by ion–molecule reaction experiments (IMR) performed in an in-house modified Ion Trap mass spectrometer (IT-MS). The kinetic analysis revealed that SO_2_ is efficiently oxidised by oxygen-atom (OAT), oxygen-ion (OIT) and double oxygen transfer (DOT) reactions. The main difference from the previously investigated free reactive ClO_2_**^−^** is the occurrence of intracluster OIT and DOT processes, which are mediated by the different ligands of the chlorite anion. This gas-phase study highlights the importance of studying the intrinsic properties of simple reacting species, with the aim of elucidating the elementary steps of complex processes occurring in solution, such as the oxidation of sulphur dioxide.

## 1. Introduction

Pollution and other environmental issues are typically associated with the atmospheric emissions of exhaust flue gases produced by power plants and industries [1]. Different technologies, collectively known as flue gas cleaning processes, attempt to mitigate the release of greenhouse gases deriving from the burning of coal to generate electrical power [2]. Most efforts in this field are aimed at planning pollutant-control strategies to reduce sulphur dioxide which is referred to as the main precursor of acid rainfalls and atmospheric particulate [3,4,5]. To this end, the European Union established the 2016/2284/UE Regulation that intends to progressively reduce SO_2_ emissions until 2029 and for the next few years [6].

Among the flue gas desulphurization (FGD) methods, the wet scrubbing system is a low-cost and simple technology based on the reaction between SO_2_ and an alkaline sorbent, typically limestone [7,8,9]. Although engineers mostly design separate air-cleaning devices for individual gas emission removal, the search for multi-pollutant control systems would reduce the need for large installation areas and operation costs [10]. To this end, sodium chlorite (NaClO_2_) is one of the most effective reagents for the simultaneous removal of oxides of sulphur (SO_x_) and nitrogen (NO_x_) [11]. The addition of this compound to seawater solution has been recently exploited to improve the elimination of SO_2_ and favour the development of environmentally friendly seawater-based FGD [12].

The strong oxidative properties of NaClO_2_ allows the conversion of sulphites (SO_3_^2−^) produced by SO_2_ absorption to the harmless sulphates (SO_4_^2−^) that are easily solubilized in water and thus removed [13,14]. Nevertheless, many factors can affect the outcome of the scrubbing process (e.g., pH, temperature, oxidant concentration, oxidant/gas contact time, volumetric gas, liquid flow rates) and the influence of these parameters has to be carefully evaluated in the design of the operating systems [15,16,17]. For instance, solution salinity is known to increase SO_2_ absorption efficiency, and under alkaline conditions needed for SO_2_/SO_3_^−^ conversion, the occurrence of a gas-solid interface reaction between SO_2_ and NaClO_2_ gives rise to the formation of Cl**^·^**, ClO**^·^** and OClO**^·^** chlorinated species which may enhance the concomitant NO oxidation in multi-pollutant removal plants [18,19]. On the other side, the above-mentioned factors can contribute to masking the intrinsic reactivity of NaClO_2_ towards sulphur dioxide preventing the elucidation of the mechanistic details that lead to the oxidation of SO_2_ and the formation of collateral products.

A successful strategy to avoid solution interfering effects and investigate the chemical processes at a strictly molecular level consists in performing gas-phase studies by mass spectrometry [20,21,22,23,24,25,26]. This technique is one of the most routinely employed for analytical purposes in a plethora of research fields spanning, inter alia, from foods and drugs to biology [27,28,29,30,31,32,33,34] or from geology to atmospheric chemistry [35,36,37,38,39]. Less well known is the use of mass spectrometry in fields such as catalysis, nevertheless, in the last years, mass spectrometry has been widely employed to assess the elementary steps of a chemical transformation by unravelling mechanistic pathways and elucidating the factors which affect the reaction outcome [40,41,42,43,44,45,46]. Accordingly, ion-molecule reaction (IMR) experiments were largely intended to investigate the reactivity of ionic reagents generated at their ground state towards neutral species under single-collision conditions. The gas-phase reaction of free ClO^−^ and ClO_2_^−^ anions towards SO_2_ has actually provided important information on the intrinsic properties of naked chlorite leading to the oxidation of sulphur dioxide to SO_3_, SO_3_**^·−^** and SO_4_**^·^^−^**, with the concomitant formation of the chlorinated species ClO^−^, ClO**^·^**, and Cl**^·^** [47]. These reaction channels, respectively referred to as oxygen-atom (OAT), oxygen-ion (OIT), and double oxygen transfer (DOT), may represent simplified models of large-scale reactions occurring in the atmosphere or involved in the flue-gas desulphurization processes.

In addition, electrospray ionization mass spectrometry has been long-time devoted to the study of salt speciation [48,49,50] showing its capability in controlling the size and charge of cluster ions. As a result, ionic clusters can be considered miniaturized systems to investigate the intrinsic features of matter aggregation phenomena [51,52]. Accordingly, the study of the gas-phase reactions of SO_2_ with positive and negative carbonate cluster ions contributed to highlighting the major role of the charge in the kinetics of smallest clusters, as well as the different reactivity when charged cluster are ligated to a NaOH molecule [53]. Indeed, a point-charge ligand can generate oriented external electric fields able to change thermodynamics and kinetics of a gas-phase thermal process by controlling the reaction mechanism, efficiencies, and product distribution [54,55,56].

Continuing with our studies focused on the chemistry of sulphur dioxide [57,58,59,60,61,62], here we report on the gas-phase reactivity of negatively charged chlorite cluster ions, [NaL·ClO_2_]^−^ (L = ClO_x_^−^ with *x* = 0–3), towards SO_2_ investigated by ion-molecule reaction experiments. In this way, the effect of the ligation of a neutral molecule to ClO_2_^−^ that changes the ion size and charge distribution of the cluster has been evaluated based on the known reactivity of naked ClO_2_^−^ species with SO_2_.

## 2. Results and Discussion

**Figure 1:**
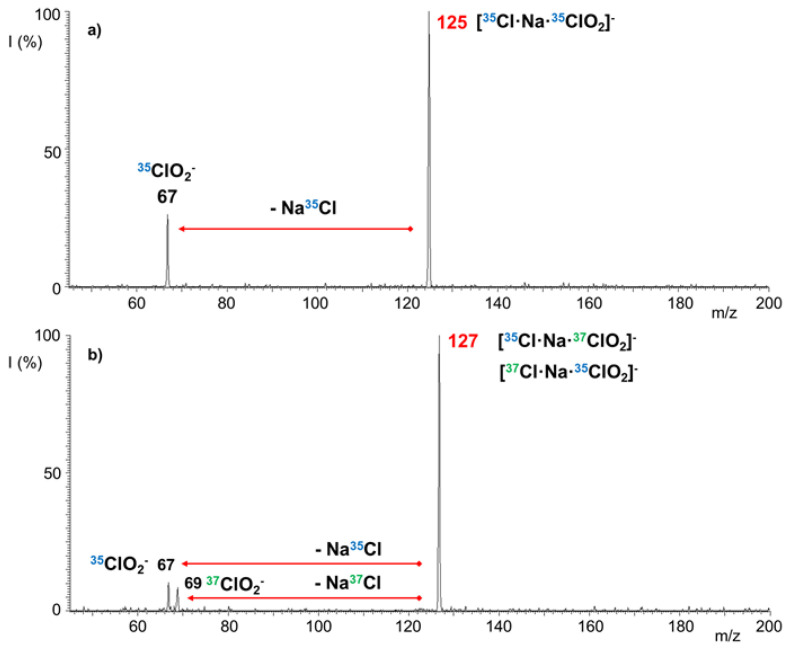
ESI-(-) CID mass spectra of the isolated (**a**) [Na^35^Cl·^35^ClO_2_]^−^ at *m*/*z* 125 and (**b**) [Na^35^Cl·^37^ClO_2_]^−^ and [Na^37^Cl·^35^ClO_2_]^−^ at *m*/*z* 127.

Oxo-halogenated ions investigated in this work were generated by the negative electrospray ionization of NaClO_2_ solutions typically yielding a series of singly-charged cluster ions in which NaClO_2_ is clustered to the ClO_2_^−^ anion to form aggregates resembling the general formula [(NaClO_2_)_n_·ClO_2_]^−^, *n* varying from 1 to 4 in the *m*/*z* range 100–500 (Figure S1). Aggregation phenomena are indeed characteristic of electrosprayed saline compounds [49] and are influenced by the solute concentrations and source parameters [53]. Furthermore, the electric field applied between the capillary and the skimmer plate accounts for the occurrence of electrochemical reactions at the conductive contact-solution interface near the ES emitter [63]. The detection of ClO_x_^−^ (*x* = 0, 1, 3) anions in addition to the ClO_2_^−^ parent species suggests the effective occurrence of in-source redox processes. For *x* = 1 and 3, the corresponding ClO^−^ and ClO_3_^−^ anions do not undergo significant aggregation phenomena. On the contrary, Cl^−^ anions promote aggregation with NaClO_2_ to form [(NaClO_2_)_n_·Cl]^−^ ions (*n* = 1–5), and mixed clusters of general formula [Na_x_Cl_y_O_z_]^−^ were also identified as minor species, as shown in the Supplementary Materials (Figure S1). The simplest ClO_2_^−^ clusters for *n* = 1 were found at *m*/*z* 125 and 157 and respectively attributed to the ^35^chlorine isotopologue of [NaCl·ClO_2_]^−^ and [NaClO_2_·ClO_2_]^−^ species. The assignment was based on the distinctive ^35/37^Cl isotope pattern and on the corresponding collision-induced dissociation (CID) mass spectra. The ion [Na^35^Cl·^35^ClO_2_]^−^ at *m*/*z* 125 predominantly fragments by losing a Na^35^Cl neutral counterpart giving rise to the ^35^ClO_2_^−^ daughter ion at *m*/*z* 67 (Figure 1a). The gas-phase decomposition of the corresponding ^35/37^Cl isotopomer (*m*/*z* 127) predictably leads to the formation of an equal ratio of ^35^ClO_2_^−^ and ^37^ClO_2_^−^ fragments at *m*/*z* 67 and 69, respectively (Figure 1b). The parent ion can be therefore described as a complex of the type [Cl·Na·ClO_2_]^−^ in which both the chloride (Cl^−^) and chlorite (ClO_2_^−^) anions are coordinated to the sodium cation (Na^+^). In particular, the chlorite moiety is reasonably consistent with an OClO^−^ species rather than with the more stable ClOO^−^ isomer, the presence of which can be excluded considering the structure of the precursor salt, NaClO_2_, and the high energy barrier to the isomerization, calculated to be 51.1 kcal·mol^−1^, [47] which cannot be overcome by the ions during the ionization process.

**Figure 2:**
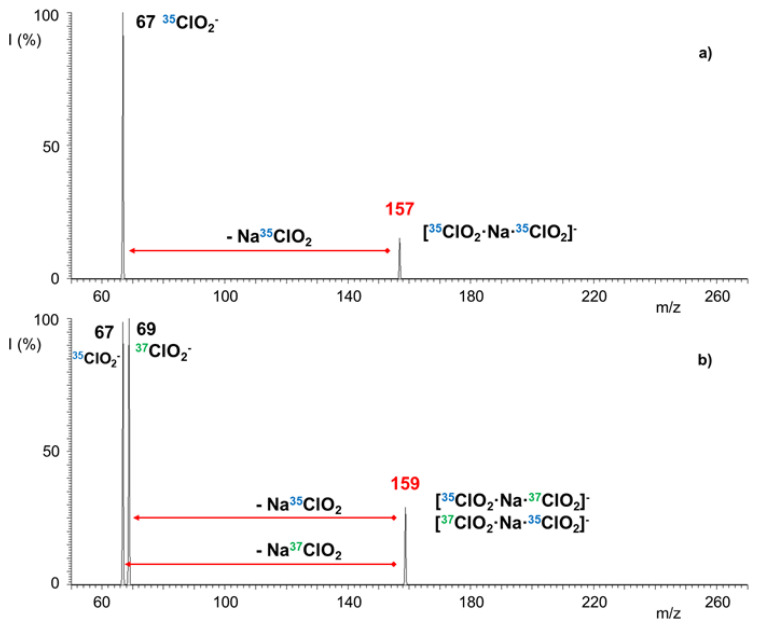
ESI-(-) CID mass spectra of the isolated (**a**) [Na^35^ClO_2_·^35^ClO_2_]^−^ at *m*/*z* 157 and (**b**) [Na^35^ClO_2_·^37^ClO_2_]^−^ and [Na^37^ClO_2_·^35^ClO_2_]^−^ at *m*/*z* 159.

Similarly, the CID mass spectrum of the ionic species [Na^35^ClO_2_·^35^ClO_2_]^−^ at *m*/*z* 157 shows the only daughter ion ^35^ClO_2_^−^ at *m*/*z* 67, arising from the loss of a NaClO_2_ neutral counterpart (Figure 2a). Accordingly, the gas-phase decomposition of the isotopomer at *m*/*z* 159 leads to the formation of an equal ratio of ^35^ClO_2_^−^ and ^37^ClO_2_^−^ fragment ions at *m*/*z* 67 and 69 (Figure 2b), accounting for the symmetrical complex [ClO_2_·Na·ClO_2_]^−^.

Each ionic species described above was in turn isolated into the ion trap and exposed to unreactive gas (He) over long accumulation times. Since no remarkable signal loss occurred, these ions can be considered rather stable gaseous chlorine-based aggregates. When reacted with SO_2_, they showed a noteworthy reactivity. In the following, the reactivity of selected cluster ions, [Cl·Na·ClO_2_]^−^ and [ClO_2_·Na·ClO_2_]^−^, will be described in depth, starting from the simplest [Cl·Na·ClO_2_]^−^. At the occurrence, the formula of the reacting species is written with the sodium cation in the centre, to highlight the reactive anionic units. Similar to the reactions observed with the non-clustered ClO_2_^−^ ions [47], both [Cl·Na·ClO_2_]^−^ and [ClO_2_·Na·ClO_2_]^−^ cluster ions promote oxygen-atom transfer (OAT), oxygen-ion transfer (OIT), and double oxygen transfer (DOT) towards SO_2_. The main difference with the free ClO_2_^−^ is that when SO_2_ is oxidised, the oxidised products predominantly remain in the cluster and are not released as free species. Accordingly, the whole mechanistic picture of the reactions between [Cl·Na·ClO_2_]^−^ and [ClO_2_·Na·ClO_2_]^−^ anions towards SO_2_ was outlined by identifying direct and consecutive pathways, measuring the rate constants for each reaction channel, and structurally characterizing the ionic products by CID experiments.

### 2.1. Reactivity of [Cl·Na·ClO_2_]^−^ Cluster Anion

**Scheme 1:**
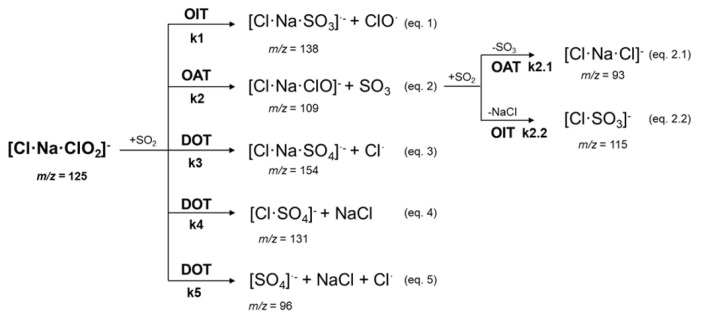
Reactivity scheme [Cl·Na·ClO_2_]^−^ ions (*m*/*z* = 125) with SO_2_. Oxygen transfer channels (OIT, OAT, DOT) and bimolecular rate constants are indicated for each reaction.

**Figure 3:**
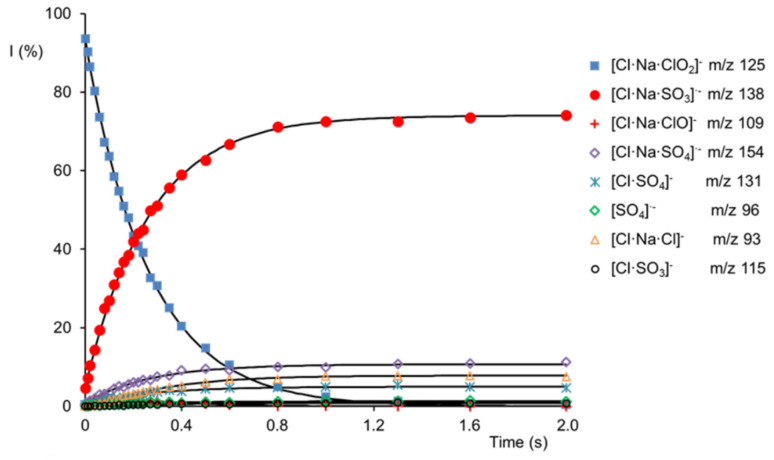
Kinetic plot and best fit lines of the reaction of isolated [NaCl·ClO_2_]^−^ ions (*m*/*z* = 125) with SO_2_. P SO_2_ = 5.26 × 10^−7^ Torr. ■ [Cl·Na·ClO_2_]^−^ (*m*/*z* 125) (R^2^ = 0.9991); ● [Cl·Na·SO_3_]**^·^**^−^ (*m*/*z* 138) (R^2^ = 0.9986); **+** [Cl·Na·ClO]^−^ (*m*/*z* 109) (R^2^ = 0.9770); ◇ [Cl·Na·SO_4_]**^·^**^−^ (*m*/*z* 154) (R^2^ = 0.9915); × [Cl·SO_4_]^−^ (*m*/*z* 131) (R^2^ = 0.9868); ◇ [SO_4_]**^·^**^−^ (*m*/*z* 96) (R^2^ = 0.9846); **△** [Cl·Na·Cl]^−^ (*m*/*z* 93) (R^2^ = 0.9946); ○ [Cl·SO_3_]^−^ (*m*/*z* 115) (R^2^ = 0.9946).

[Cl·Na·ClO_2_]^−^ cluster anions react with SO_2_ at room temperature giving rise to the products shown in Scheme 1, through a complex series of parallel and consecutive reactions. A kinetic plot showing the time progress of the reaction is displayed in Figure 3. The identity of the ionic products from reactions 1–5 has been probed by collision-induced dissociation as discussed in the following. As reported in Table 1, the reaction of [Cl·Na·ClO_2_]^−^ has a rate constant (k_dec_) of 2.88 × 10^–10^ (±30%) cm^3^ s^−1^ mol^−1^ and an efficiency (k/k_coll_) of 24.2%.

Although the larger size of [Cl·Na·ClO_2_]^−^ is predictably responsible for the decrease of the overall reaction rate compared to that of naked ClO_2_^−^ (2.88 vs. 9.10 × 10^−10^ cm^3^ s^−1^ mol^−1^), the intrinsic reactivity of the two ionic species is comparable, except for small differences in the branching ratios of the three oxygen transfer reactions. For the sake of clarity, the reactivity, OIT, OAT and DOT, is indicated in each reaction channel.

The main reaction of [Cl·Na·ClO_2_]^−^ leads to the formation of the ionic product [Cl·Na·SO_3_]**^·^**^−^ at *m*/*z* 138 and a ClO**^·^** radical species (Equation (1)). The reaction proceeds quickly with a rate constant k_1_ of 2.13 × 10^−10^ (±30%) cm^3^ s^−1^ mol^−1^ (Table 2), and a branching ratio of 74.2% (Table 1). The collision-induced dissociation of the product ion at *m*/*z* 138 gives rise to the SO_3_^−^ ion at *m*/*z* = 80 (Figure S3) through the loss of a neutral NaCl consistent with a [Cl·Na·SO_3_]**^·^**^−^ connectivity, hinting at the occurrence of an intracluster oxidation of SO_2_ to SO_3_^−^ through an oxygen ion transfer (OIT) process.

Furthermore, [Cl·Na·SO_3_]**^·^**^−^ was found to be unreactive towards SO_2_ thus confirming the presence of the two notoriously inert Cl^−^ and SO_3_**^·^^−^** moieties [47]. The Cl^−^ anion only plays a spectator role, whereas the sodium cation is reasonably involved in the coordination of both Cl^−^ and SO_3_**^·^**^−^ anions.

A minor channel gives rise to [Cl·Na·ClO]^−^ at *m*/*z* 109 and SO_3_ (Equation (2)), with a rate constant k_2_ of 2.51 × 10^−11^ (±30%) cm^3^ s^−1^ mol^−1^ (Table 2), and a branching ratio of 8.8% (Table 1). The product ion [Cl·Na·ClO]^−^ at *m*/*z* 109 resembles an aggregate in which a Cl^−^ spectator anion and a ClO^−^ moiety are both coordinated to sodium cation, as evidenced by its CID mass spectrum. Through this path, SO_2_ is therefore oxidised to SO_3_ by an oxygen atom transfer (OAT) reaction. Once formed, [Cl·Na·ClO]^−^ displays the distinctive reactivity of the surrounding ClO^−^ moiety towards SO_2_ [47] that consists in a further SO_2_ to SO_3_ conversion (Equation (2.1)), through a second OAT process, and in an intracluster reaction giving [Cl·SO_3_]^−^ at *m*/*z* = 115 through an OIT process (Equation (2.2)). The rate constants of the two competitive processes are respectively k_2.1_ = 7.53 × 10^−10^ (±30%) and k_2.2_ = 7.43 × 10^−11^ (±30%) cm^3^ s^−1^ mol^−1^ (Table 2). Not surprisingly, the OAT undergone by [Cl·Na·ClO_2_]^−^ (Equation (2)) is slower with respect to the same process undergone by [Cl·Na·ClO]^−^ (Equation (2.1)), reflecting the different reactivity of the free ClO_2_^−^ and ClO^−^ species [47]. The first preferably oxidises SO_2_ through an OIT process, whereas the OAT is faster in the case of ClO^−^.

Finally, the [Cl·Na·ClO_2_]^−^ parent ion is involved in different reactions collectively responsible for a double oxygen transfer (DOT) to SO_2_ with the formation of product ions containing a sulphate anion, SO_4_**^·^**^−^ (Equations (3)–(5)). The sulphate moiety can be either found as a clustered ion, as in Equations (3) and (4), or it can be a free anion as in Equation (5). In Reactions (3) and (4), upon the oxidation of SO_2_ to SO_4_**^·^**^−^, a NaCl or Cl^·^ neutral moieties are respectively released. In any case, it is formed through an overall O_2_^−^ transfer and the DOT processes account for a branching ratio of 17.0% (Table 1).

The ionic product at *m*/*z* 154 (Equation (3)) is consistent with a [Cl·Na·SO_4_]^−^ structure according to its fragmentation into SO_4_**^·^**^−^ ion at *m*/*z* = 96 (Figure S4) and loss of the neutral NaCl. The reaction occurs with a rate constant k_3_ of 3.06 × 10^−11^ (±30%) cm^3^ s^−1^ mol^−1^ and represents the main DOT path. Alternatively, the SO_4_**^·^**^−^ moiety can remain attached to the Cl^·^ radical, and releasing a NaCl moiety leads to the product ion [Cl·SO_4_]^−^ at *m*/*z* 131 with a k_4_ of 1.47 × 10^−11^ (±30%) cm^3^ s^−1^ mol^−1^ (Equation (4)). According to the electron affinity values for SO_4_ (EA = 5.10 eV) and Cl (EA = 3.61 eV) [64], the negative charge of the [Cl·SO_4_]^−^ product ion is mostly located on the SO_4_ moiety, as confirmed by the dissociation of this cluster into SO_4_**^·^**^−^ ion at *m*/*z* = 96 (Figure S5).

Finally, SO_4_**^·^**^−^ is also generated as a free ion through reaction 5 with a k_5_ of 3.4 × 10^−12^ (±30%) cm^3^ s^−1^ mol^−1^. Not surprisingly, the free SO_4_**^·^**^−^ ion is the least abundant product formed through the DOT paths. In the clustered species, [Cl·SO_4_]^−^ and [Cl·Na·SO_4_]**^·^**^−^, the negative charge can be more favourably dispersed in larger species.

The comparison with the reactivity of the free ClO_2_^−^ anion shows that also with the [Cl·Na·ClO_2_]^−^ clustered anions the OIT remains the main reaction channel. When ClO_2_^−^ was reacted with SO_2_, the small differences in the electron affinities between ClO**^−^** (EA of 2.27 eV) and SO_3_ (EA of 2.06 eV) [64] only resulted in close energies (−24.6 and −25.6 kcal mol^−1^) calculated for the two alternative exit channels, namely SO_3_**^·^**^−^ (+ClO**^·^**) and SO_3_ (+ClO^−^) [47]; therefore, the prevalence of the OIT process was attributed to kinetic factors. Contrarywise, thermochemical factors favoured the OAT reaction over the OIT process in the reactivity of the free ClO^−^ anion with SO_2_ due to the significantly higher electron affinity of Cl (EA = 3.61 eV) with respect to that of SO_3_ (EA = 2.06 eV). Accordingly, in the reactivity of [Cl·Na·ClO]^−^ ion, OAT (Equation (2.1)) prevails over the OIT process (Equation (2.2)) by a ratio of ca. 10/1. The in-depth theoretical analyses performed on the free ClO_2_^−^ species [47] can also give some insights into the reactivity observed with the clustered chlorite anions. The potential energy surface (PES) of [OClO-SO_2_]^−^ system, was characterised by an early transition state that accounts for the almost barrierless formation of SO_3_**^·^**^−^. In the TS, the negative charge is exclusively located on the preformed SO_3_ group (1.02 e^−^), that is prone to rapid dissociation into the sulphite radical anion, and that strongly competes with the OAT and DOT processes. The formation of SO_3_ and SO_4_**^·^**^−^ occurs through common intermediates, found on the double well PES, which dissociate reflecting the endothermicity of the two processes. This theoretical analysis is well suited to also explain the reactivity observed for the ligated [Cl·Na·ClO_2_]^−^ cluster ions with SO_2_, and that of the other ligated species described in the following sections. The NaCl ligand does not affect the outcome of the oxidation reactions. Rather, it seems to have the effect of spreading the charge on the cluster, eventually lowering the reaction rate.

Overall, an increase of DOT and OAT processes at the expense of OIT channel is evidenced for the [Cl·Na·ClO_2_]^−^ cluster ion with respect to the non-clustered ClO_2_^−^ anion.

#### Effect of the Ligand

To deeply investigate the role of the NaCl ligand in the reactivity of [Cl·Na·ClO_2_]^−^ ion towards SO_2,_ Cl^−^ was first replaced by X anion (X = F, Br, I) to form the corresponding [X·Na·ClO_2_]^−^ reactive species and subsequently Li^+^ was inserted in place of Na^+^ to evaluate the role of the cation. Only non-redox-active ligands were used in order to make a comparison with the effect of salinity in solution, where it is known that the increase in ionic strength determines an increase in the absorption efficiency of SO_2_, [18].

As reported in Table 1, the overall rate constant (k_dec_) increases with the charge density of X anion, reaching the highest value of 3.75 × 10^−10^ (±30%) cm^3^ s^−1^ mol^−1^ with the smallest F anion (ion radius = 136 pm) and the lowest value of 1.85 × 10^−10^ (±30%) cm^3^ s^−1^ mol^−1^ with the largest I anion (ion radius = 216 pm) (Table 1) [65].

Regarding the branching ratios of the three reaction channels, [I·Na·ClO_2_]^−^ and [Br·Na·ClO_2_]^−^ show a reactivity distribution comparable to that of the [Cl·Na·ClO_2_]^−^ ion. On the contrary, an increase of the OIT process at the expense of the OAT and DOT channels was reported for [F·Na·ClO_2_]^−^ cluster species. As a consequence of the high charge density on the fluoride ion, the [F·Na·SO_3_]**^·^**^−^ ionic product arising from the OIT process adds an SO_2_ molecule giving rise to a labile adduct of the type [F·Na·SO_3_·SO_2_]**^·^**^−^ never observed with the other reactant ions. The OAT process increases down the halogen series, whereas the opposite occurs with the OIT process.

Passing to the cation effect, an opposite trend was observed, as the reaction rate decreases by increasing the positive charge density on the metal. The overall rate constant for the [Cl·Li·ClO_2_]^−^ + SO_2_ reaction is indeed almost three times lower than the corresponding value for the [Cl·Na·ClO_2_]^−^ + SO_2_ system, highlighting the central role of an external electric field in modelling the reaction kinetics. [54,55,56] Minor effect of the charge density is instead reported for the general reactivity scheme of [Cl·Li·ClO_2_]^−^ anion.

The main role played by the different ligands described above might be due to the spreading the negative charge of ClO_2_^−^ within the cluster, the effect of which is reflected in the oxidative capacity of ClO_2_^−^ ion: the higher the charge density of the ligand, the faster the reaction. Passing from F^−^ to I^−^, the former forms a tighter ion pair with Na^+^, making the chlorite anion more available to oxidise sulphur dioxide. A second effect concerns the steric hindrance to the approach of SO_2_ due to the neutral ligand, whereby ligands of larger dimensions lead to a decrease in the overall reaction rate. An opposite effect is observed with the lithium cation, whose small size increases the interactions with both Cl^−^ and ClO_2_^−^, reducing the oxygen transfer rate of the latter. The effect of the non-redox ligands here tested is different to that played in solution, where an increase in ionic strength (i.e., the salinity) has the effect of increasing the SO_2_ absorption efficiency by the solutions and therefore the overall efficiency of wet scrubbing processes [18].

### 2.2. Reactivity of [ClO_2_·Na·ClO_2_]^−^ Cluster Anion

**Scheme 2:**
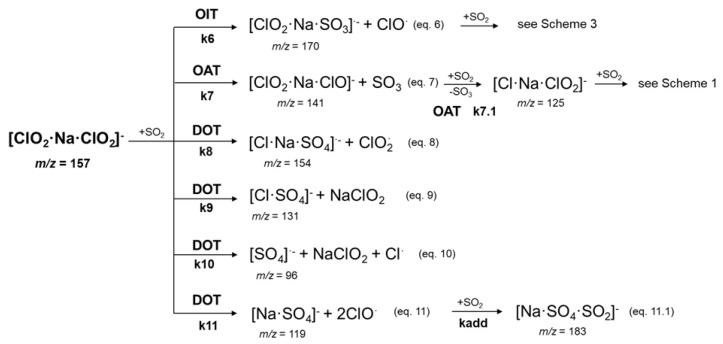
Reactivity scheme [ClO_2_·Na·ClO_2_]^−^ ions (*m*/*z* = 157) with SO_2_. Oxygen transfer channels (OIT, OAT, DOT) and bimolecular rate constants are indicated for each reaction.

Passing to [ClO_2_·Na·ClO_2_]^−^ cluster ion, the reactive channels reported in Scheme 2 have been observed from the reaction with SO_2_. The identity of the ionic products from Reactions (6)–(11) have been probed by collision−induced dissociation experiments as discussed below.

**Figure 4:**
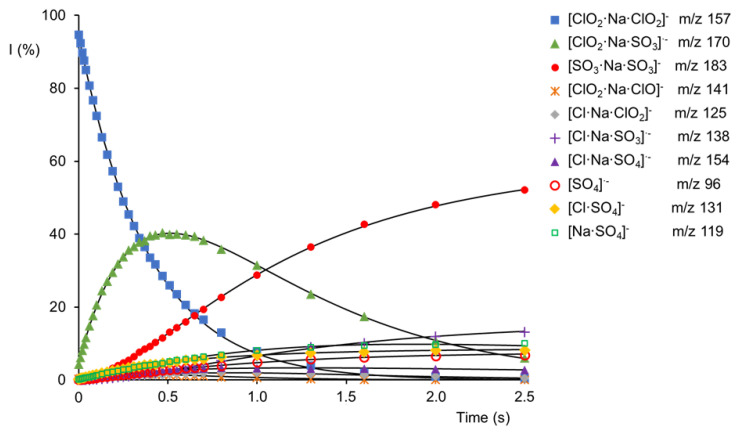
Kinetic plot and best fit lines of the reaction of isolated [NaClO_2_·ClO_2_]^−^ ions (*m*/*z* = 157) with SO_2_. P SO_2_ = 3.30 × 10^−7^ Torr. ■ [ClO_2_·Na·ClO_2_]^−^ (*m*/*z* 157) (R^2^ = 0.9991); ▲[ClO_2_·Na·SO_3_]**^·^**^−^ (*m*/*z* 170) (R^2^ = 0.9986); ● [SO_3_·Na·SO_3_]^−^ (*m*/*z* 183) (R^2^ = 0.9998); **×** [ClO_2_·Na·ClO]^−^ (*m*/*z* 141) (R^2^ = 0.9888); ♦ [Cl·Na·ClO_2_]^−^ (*m*/*z* 125) (R^2^ = 0.9965); **+** [Cl·Na·SO_3_]^·−^ (*m*/*z* 138) (R^2^ = 0.9967); ▲ [Cl·Na·SO_4_]^·−^ (*m*/*z* 154) (R^2^ = 0.9912); **○** [SO_4_]^·−^ (*m*/*z* 96) (R^2^ = 0.9946); ♦ [Cl·SO_4_]^−^ (*m*/*z* 131) (R^2^ = 0.9992); □ [Na·SO_4_]^−^ (*m*/*z* 119) (R^2^ = 0.9971).

The reaction of [ClO_2_·Na·ClO_2_]^−^ with SO_2_ at 298 K is fast and efficient showing an overall rate constant (k_dec_) of 7.48 × 10^−10^ (±30%) cm^3^ s^−1^ mol^−1^ at 298 K. This value is only 0.82 times lower than the corresponding one for the bare ClO_2_^−^ species, whereas the efficiencies (k/k_coll_) of the two processes are similar (66.0% vs. 63.8%, Table 1). The intrinsic reactivity of the [ClO_2_·Na·ClO_2_]^−^ anion towards SO_2_ is comparable to that of the [Cl·Na·ClO_2_]^−^ species, as demonstrated by rather close branching ratios for the three reaction pathways (Table 1). Nonetheless, the concomitant presence of two reactive ClO_2_^−^ moieties in the [ClO_2_·Na·ClO_2_]^−^ ion gives rise to an intricate reaction picture as shown in the above Scheme 2 and in the kinetic plot of Figure 4.

The main reaction of [ClO_2_·Na·ClO_2_]^−^ ion at *m*/*z* 157 leads to the ionic product at *m*/*z* 170, attributed to [ClO_2_·Na·SO_3_]**^·^**^−^, and a ClO**^·^** radical species (Equation (6)). The CID mass spectrum of the ionic product at *m*/*z* 170 shows a major dissociation into SO_3_**^·^**^−^, which accounts for a [ClO_2_·Na·SO_3_]**^·^**^−^ structure (Figure S7). As in the case of Cl-clustered species [Cl·Na·ClO_2_]^−^, the main reaction of [ClO_2_·Na·ClO_2_]^−^ consists of an oxygen ion transfer, resulting in a fast intracluster oxidation of SO_2_. The rate constant k_6_ is 6.26 × 10^−10^ (±30%) cm^3^ s^−1^ mol^−1^ (Table 3), and a branching ratio of 81.8% (Table 1). The intracluster formation of SO_3_**^·^**^−^ gives rise to a negatively charged product in which one of the two ClO_2_^−^ moieties only played a spectator role, whereas the sodium cation is reasonably involved in the coordination of the ClO_2_^−^ and SO_3_**^·^**^−^ anions. However, the presence of a residual ClO_2_^−^ moiety in the product ion [ClO_2_·Na·SO_3_]**^·^**^−^ is responsible for the consecutive reactivity of this species, which is deeply discussed in the next paragraph (vide infra). The complete reactive scheme of [ClO_2_·Na·ClO_2_]^−^, integrated with the reactivity of [ClO_2_·Na·SO_3_]**^·^**^−^, is reported in the Supplementary Materials (Scheme S1), showing the complex and intricate reactivity of an only apparently simple species.

A second path, indeed a minor one, leads to an ionic product at *m*/*z* 141 and SO_3_ (Equation (7)), formed through an OAT from one of the two ClO_2_^−^ unit to SO_2_. The branching ratio is only 4.8% (Table 1) and a rate constant k_7_ of 3.64 × 10^−11^ (±30%) cm^3^ s^−1^ mol^−1^ (Table 3). The ionic product at *m*/*z* 141, resembles an aggregate in which a ClO_2_^−^ anion and a ClO**^−^** reactive moiety are both coordinated to sodium cation, although its fragmentation into the ClO_3_^−^ species at *m*/*z* 83 seems to account for a [Cl·Na·ClO_3_]^−^ structure (Figure S8). Nonetheless, the [Cl·Na·ClO_3_]^−^ ion obtained by spraying a NaCl/NaClO_3_ (1:1) millimolar solution resulted to be not reactive towards SO_2_ (Figure S9).

Therefore, it seems more likely to attribute the [ClO·Na·ClO_2_]^−^ connectivity to the ion at *m*/*z* 141 that rearranges to [Cl·Na·ClO_3_]^−^ upon CID, thus demonstrating the interaction of sodium cation with the ClO_2_^−^ and ClO^−^ anions, rather than Cl^−^ and ClO_3_^−^ species. The presence of two potential reactive units, ClO and ClO_2_, make the [ClO·Na·ClO_2_]^−^ cluster ion quite reactive. The consecutive OAT process observed (Equation (7.1)) has a rate constant k_7.1_ of 9.57 × 10^−10^ (±30%) cm^3^ s^−1^ mol^−1^ (Table 3), which is much higher than k_7_ relative to the similar OAT process in Equation (7). Again, as for reactions 2 and 2.1, the reason lies in the different reactivity of the free chlorite and hypochlorite anions, the first undergoing faster OIT and the second faster OAT processes. The product ion at *m*/*z* 125 corresponds to the [Cl·Na·ClO_2_]^−^ species, as demonstrated by its characteristic fragmentation pattern and the distinctive reactivity discussed in the previous paragraph (Figures S10 and S11).

Four different DOT channels were reported for the [ClO_2_·Na·ClO_2_]^−^ parent ion. The first three pathways (Equations (8)–(10)) resemble those already described for the [Cl·Na·ClO_2_]^−^ ion (Equations (3)–(5)), as the same product ions at *m*/*z* 154, 131, and 96 are respectively detected. The intracluster DOT processes reported in Equations (3) and (8) occurs with similar rate constants k_3_ and k_8_, regardless the ligand [NaCl] or [NaClO_2_] attached to the ClO_2_^−^ moiety (k_3_ = 3.06 vs. k_8_ = 2.51 × 10^−11^ cm^3^ s^−1^ mol^−1^). A significantly faster formation of the [Cl·SO_4_]^−^ ion at *m*/*z* 131 was reported for the [ClO_2_·Na·ClO_2_]^−^ parent species with respect to [Cl·Na·ClO_2_]^−^ (k_9_ = 4.17 vs. k_4_ = 1.47 × 10^−11^ cm^3^ s^−1^ mol^−1^). Again, the formation of the free [SO_4_]**^·^**^−^ product ion represents the lowest DOT process (k_10_ = 1.34 × 10^−11^ cm^3^ s^−1^ mol^−1^). In addition, a fourth DOT channel was observed only for the [ClO_2_·Na·ClO_2_]^−^ parent ion which is worthy of note. In this case, the oxidation of SO_2_ leads to the product at *m*/*z* 119 with a rate constant k_11_ of 2.22 × 10^−11^ cm^3^ s^−1^ mol^−1^ (Equation (11)) which subsequently adds a further SO_2_ molecule with a k_add_ of 5.74 × 10^−11^ cm^3^ s^−1^ mol^−1^ (Equation (11.1), Table 3). Although the structure of the ionic species at *m*/*z* 119 could not be directly probed owing to its unproductive CID, a possible [Na·SO_4_]^−^ formula can be reasonably supposed. The corresponding ion at *m*/*z* 119 was also obtained by electrospraying a solution of Na_2_SO_4_ which, once isolated and reacted with SO_2_, gave a ligated [Na·SO_4·_SO_2_]^−^ addition product with a rate constant consistent with k_add_, of Equation (11.1), thus confirming the identity of the parent species at *m*/*z* 119 (Figure S12). The [Na·SO_4_]^−^ formula accounts for the oxidation of the sulphur atom of sulphur dioxide and the eventual reduction of the chlorine atoms of the [ClO_2_·Na·ClO_2_]^−^ ion. Both the ClO_2_^−^ units may be involved in the reaction, in which each ClO_2_^−^ anion transfers an O^·−^ moiety to SO_2_ giving rise to an SO_4_^2−^ species and the release of two ClO^−^ radicals. This hypothesis was confirmed by replacing one of the two ClO_2_^−^ anions with the similarly oxygenated, but intrinsically unreactive ClO_3_^−^ anion to obtain the [ClO_3_·Na·ClO_2_]^−^ parent ion. When exposed to SO_2_, this species shows an intrinsic reactivity comparable to that of the [ClO_2_·Na·ClO_2_]^−^ ion with the only exception of the product at *m*/*z* 119 that was not observed, thus highlighting the involvement of both ClO_2_^−^ anions in the double O**^·^**^−^ transfer.

### 2.3. Reactivity of [SO_3_·Na·ClO_2_]**^·^**^−^ Cluster Anion

**Scheme 3:**
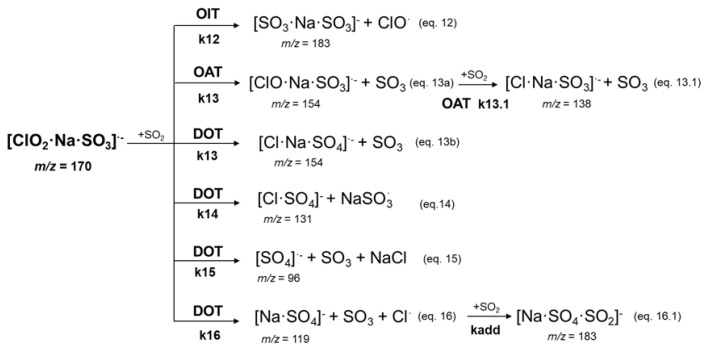
Reactivity scheme [ClO_2_·Na·SO_3_]**^·^**^−^ ions (*m*/*z* = 170) with SO_2_. Oxygen transfer channels (OIT, OAT, DOT) and bimolecular rate constants are indicated for each reaction.

To better investigate the consecutive reactivity of the product ion at *m*/*z* 170, arising from the [ClO_2_·Na·ClO_2_]^−^ parent species through Equation (6), the putative [ClO_2_·Na·SO_3_]**^·^**^−^ ion was isolated from the sequence 157 to 170 (MS^2^-isolated) and separately reacted with SO_2_. The reactivity observed is illustrated in Scheme 3.

**Figure 5:**
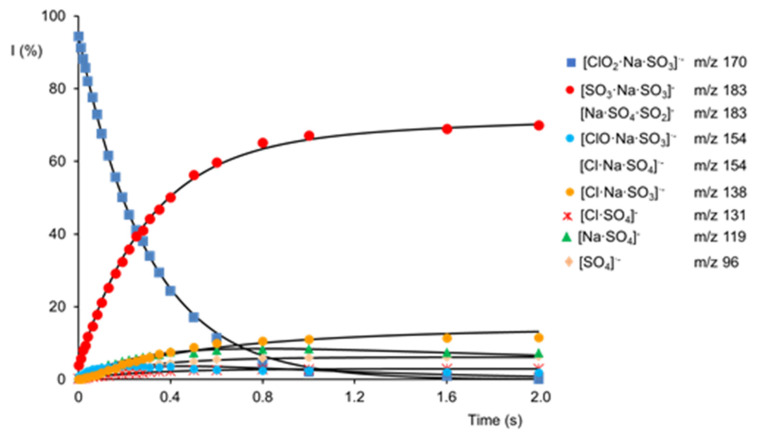
Kinetic plot and best fit lines of the reaction of isolated [ClO_2_·Na·SO_3_]**^·^**^−^ ions (*m*/*z* = 170) with SO_2_. P SO_2_ = 2.60∙× 10^−7^ Torr. ■ [ClO_2_·Na·SO_3_]**^·^**^−^ (*m*/*z* 170) (R^2^ = 0.9991); ● [SO_3_·Na·SO_3_]^−^/[Na·SO_4_·SO_2_]^−^ (*m*/*z* 183) (R^2^ = 0.9986); ● [ClO·Na·SO_3_]**^·^**^−^ / [Cl·Na·SO_4_]**^·^**^−^ (*m*/*z* 154) (R^2^ = 0.9686); ● [Cl·Na·SO_3_]**^·^**^−^ (*m*/*z* 138) (R^2^ = 0.9880); **×** [Cl·SO_4_]^−^ (*m*/*z* 131) (R^2^ = 0.9950); ▲ [Na·SO_4_]^−^ (*m*/*z* 119) (R^2^ = 0.9973); ♦ [SO_4_]**^·^**^−^ (*m*/*z* 96) (R^2^ = 0.9946).

The overall reaction shows a rate constant (k_dec_) of 3.74 × 10^−10^ (±30%) cm^3^ s^−1^ mol^−1^ and an efficiency of 33.0% at 298 K (Table 1). These values agree with those reported for the other [X·Na·ClO_2_]^−^ parent species analysed and having a unique ClO_2_^−^ reactive moiety (Table 1). Regarding, instead, the distribution of the three reaction paths, an even more pronounced increase of the DOT channels, accounting for a total amount of 22.7%, was observed (Table 1). The time progress of the reaction is described by the kinetic plot in Figure 5 and the rate constants of each pathway are reported in Table 4.

The intracluster OIT process (Equation (12)) proceeds quickly, showing a k_12_ of 2.63 × 10^−10^ (±30%) cm^3^ s^−1^ mol^−1^ (Table 4) and leading to the formation of an ion at *m*/*z* 183. Unfortunately, the CID mass spectrum of this species does not allow to distinguish between a [SO_3_·Na·SO_3_]^−^ or a [Na·SO_4·_SO_2_]^−^ structure (Figure S14), the latter already observed as a product of the DOT process involving the [ClO_2_·Na·ClO_2_]^−^ parent ion. Nonetheless, based on the reactivity of naked ClO_2_^−^ and knowing that the SO_3_**^·^**^−^ moiety is notoriously unreactive with SO_2_, it is reasonable to suppose a [SO_3_·Na·SO_3_]^−^ general formula for this species.

The [ClO_2_·Na·SO_3_]**^·^**^−^ parent ion is also involved in an OAT reaction proceeding with a k_13_ of 2.60 × 10^−11^ (±30%) cm^3^ s^−1^ mol^−1^ and giving rise to an ion at *m*/*z* 154 that is consistent with a [ClO·Na·SO_3_]**^·^**^−^ structure. The consecutive OAT reactivity of this species leading to the ion at *m*/*z* 138 (Equation (13.1); k_13.1_ = 1.09 × 10^−10^ (±30%) cm^3^ s^−1^ mol^−1^, Table 4, Figure S11) accounts for the presence of the surrounding reactive ClO^−^ moiety in [ClO·Na·SO_3_]**^·^**^−^ (*m*/*z* = 154). When MS^3^-isolated into the ion trap by the sequence 170 to 154 and exposed to SO_2_, the ionic species at *m*/*z* 154 is only partially reactive towards this neutral gas. A portion of the ionic population at *m*/*z* 154 survives over time, hinting at the concomitant presence of the unreactive [Cl·Na·SO_4_]**^·^**^−^ species together with the [ClO·Na·SO_3_]**^·^**^−^ isobaric ion that is consumed in the consecutive reaction (Figure S15). The [Cl·Na·SO_4_]**^·^**^−^ species can reasonably arise from a direct intracluster DOT channel (Equation (13b)), as previously observed in analogous processes involving the [Cl·Na·ClO_2_]^−^ and [ClO_2_·Na·ClO_2_]^−^ parent ions (Equations (3) and (8)). As a result, the O_2_^−^ transfer from ClO_2_^−^ to SO_2_ triggers the release of a neutral SO_3_ moiety according to the electron affinity values of the species involved in the reaction [27,28,29,30,31,32,33,34,65]. Unfortunately, it was not possible to independently measure k_13b_, which is therefore included with that of the OAT transfer, k_13_. As a consequence, the branching ratio of the OAT might be slightly overestimated, at the expense of that for the DOT process, which could therefore be underestimated. Two other DOT pathways reported in Equations (14) and (15) were also previously observed for the [Cl·Na·ClO_2_]^−^ and [ClO_2_·Na·ClO_2_]^−^ parent clusters (Equations (4) and (9), Equations (5) and (10)). All these pathways show rather similar formation rate constants of the 10^−11^ cm^3^ s^−1^ mol^−1^ order of magnitude (Table 4).

[ClO_2_·Na·SO_3_]**^·^**^−^ also reacts with SO_2_, leading to the [Na·SO_4_]^−^ product ion at *m*/*z* 119 (Equation (16)). The reaction, showing a k_16_ of 4.57 × 10^−11^ cm^3^ s^−1^ mol^−1^, proceeds with an intracluster O_2_^−^ transfer. Such unusual reactivity probably involves both the ClO_2_^−^ anion that triggers a classic O_2_^−^ transfer and the SO_3_**^·^**^−^ moiety that may be responsible for an electron transfer, giving rise to an SO_4_^2−^ anion through a concerted rearrangement. As previously reported (Equation (11.1)), the consecutive addition of an SO_2_ molecule to the [Na·SO_4_]^−^ product ion is observed, thus confirming the identity of the ion at *m*/*z* 119.

Finally, as to the reactivity of higher species such as [(NaClO_2_)_n_·ClO_2_]^−^, only the rate constant relative to cluster with *n* = 2 has been measured (Table 1), which does not appear to be affected by the number of additional NaClO_2_ units compared to [ClO_2_·Na·ClO_2_]^−^. However, it was not possible to evaluate the branching ratio of the OIT, OAT and DOT processes of [(NaClO_2_)_2_·ClO_2_]^−^, due to the low intensity signals relative to parent cluster ions, and to the complex array of peaks resulting from the reaction with SO_2_.

## 3. Materials and Methods

Mass spectrometric experiments were carried out on an LTQ-XL linear ion-trap mass spectrometer (Thermo Fisher Scientific) that was in-house modified to perform ion-molecule reactions (IMR) [53]. Water-acetonitrile (1:1) solutions of NaClO_2_ at millimolar concentrations were injected into the ESI (electrospray ionization) source of the instrument at a flow rate of 5 μL min^−1^ via the on-board syringe pump and using nitrogen as sheath and auxiliary gas (flow rate = 11 and 2 arbitrary units respectively, a. u. ~0.37 L min^−1^). Other [ML·ClO_2_]^−^ cluster anions (L = F, Br, I, ClO_3_; M = Li, Na) investigated in this work were obtained from millimolar solutions of 1:1 ML and NaClO_2_ salts dissolved in water-acetonitrile (1:1). To generate chlorite cluster ions and optimize the ion transmission, spray voltage was tuned in the 1.8–3.2 kV range, whereas the capillary temperature was set at 275 °C. The distribution of the ionic aggregates strictly depends on the capillary and tube lens voltage. Hence, these parameters were in turn optimized to increase the signal intensity of the parent ion under investigation.

Once generated, reagent ions were transferred to the vacuum region of the trap, mass-to-charge isolated and reacted with sulphur dioxide. Each reaction product was then mass selected by a further step of isolation, that is typical of MS^n^ experiments performed by Ion Trap mass spectrometers, and the consecutive reactivity of these species was probed towards SO_2_ to unravel a complete reaction picture. Furthermore, the ionic reactants and products were structurally characterized by collision-induced dissociation (CID) experiments performed by increasing the energy of mass-selected ions in the presence of helium as collision gas (pressure of ca. 3 × 10^−3^ Torr). Depending on the species of interest, normalized collision energies ranging between 20% and 40% were typically applied with an activation time of 30 ms. Ions were isolated with a window of 1 *m*/*z*, and the Q value was optimized to ensure stable trapping fields for all the ionic species under investigation.

Sulphur dioxide was introduced into the trap through a deactivated fused silica capillary that enters the vacuum chamber from a 6.25 mm hole placed in the backside of the mass spectrometer. The pressure of the neutral gas was kept constant by a metering valve and measured by a Granville-Phillips Series 370 Stabil Ion Vacuum Gauge. Owing to the position of the Pirani gauge, the actual sulphur dioxide pressure was estimated after calibration of the pressure reading [66]. Typical pressures of SO_2_ ranged between 1.1 × 10^−7^ and 8.0 × 10^−7^ Torr, the uncertainty was estimated to be ± 30%. The signals of the ionic reactant and products were monitored over time as a function of the neutral concentration and for each reaction time an average of 10 scan acquisitions was recorded. The normalized collision energy was set to zero and the activation *Q* value was optimized to ensure stable trapping fields for all the ions. Xcalibur 2.0.6 software was used to acquire all the displayed mass spectra.

All the reactions can be regarded as pseudo-first-order processes due to the excess of neutral gas relative to the reactant ion in the trap. DynaFit4 software package [67] was used to perform nonlinear least squares regression to simultaneously fit reactant and products concentration versus time. Experimental data from the kinetic analyses were fitted to a mathematical model consistent with the postulated reaction mechanism. To check the validity of the kinetic schemes, the obtained unimolecular rate constants were used to simulate the time progress of the reactions using the kinetic simulation function contained in DynaFit4. Bimolecular rate constants *k* (cm^3^ molecule^−1^ s^−1^) were obtained dividing the pseudo-first-order constants (s^−1^) by the concentration of neutral reagent gas. The branching ratios between the three channels (OIT, OAT, DOT) were calculated from the constants of formation of the primary direct products for each reactive species. The reaction efficiency was calculated as the ratio of the bimolecular rate constant *k* to the collision rate constant (*k_coll_*), according to the average dipole orientation (ADO) theory [68]. To ensure the accuracy of the *k* values, approximately 15 independent measurements for each precursor ion were performed on different days over a sevenfold neutral pressure range. The standard deviation in the fitting parameters of the kinetic modelling used is usually evaluated between 10–20%, whereas the uncertainty attached to the measurement of the neutral pressure is typically evaluated ±30%.

## 4. Conclusions

Mass spectrometry has been used to elucidate the gas-phase reactivity of [NaL·ClO_2_]^−^ cluster anions (L = ClO_x_^−^ with *x* = 0–3) with sulphur dioxide. These charged species can be taken as simplified models of large-scale reactions occurring in solution or in the flue-gas desulphurization processes, which are accomplished by sodium chlorite solutions.

The kinetic analysis has shown that SO_2_ was efficiently oxidised by oxygen atom transfer (OAT), oxygen ion transfer (OIT), and double oxygen transfer (DOT) respective to SO_3_, SO_3_**^·^**^−^ and SO_4_**^·^**^−^. In the case of OIT and DOT processes, an intracluster reaction was observed, by which the oxidised ionic forms of SO_2_, namely SO_3_**^·^**^−^ and SO_4_**^·^**^−^, remain within the cluster and are not released as a free species. The results here reported show that when ClO_2_^−^ is ligated to a non−redox active molecule, the complexation leads to a moderate reduction in the rate of oxidation processes, without substantially influencing the branching ratio. This effect contrasts, but not surprisingly, with what is observed in solution, where dissolved salts increase the SO_2_ capture by increasing ionic strength of the solutions. In the gas phase, the direct and strong interaction of the chlorite anion with the ligand is detrimental to the reaction rate. However, the effect of redox active ligands, metallic or metal-free, could be quite different, as suggested by the reactivity observed with [ClO_2_·Na·ClO_2_]^−^, in which the second reactive ClO_2_^−^ moiety succeeds in increasing the rate of the oxidation. Therefore, the ligation with a redox active group, different from the chlorite one, could succeed in tuning the oxidation processes.

## Figures and Tables

**Table 1:** Rate constants (cm^3^ s^−1^ mol^−1^), branching ratios (%Σ) and efficiencies (k/k_coll_) for the reactions of [L·M·ClO_2_]^−^ (L = Cl, F, Br, I, ClO_2_, SO_3_; M = Na, Li) anions with SO_2_. The reaction of ClO_2_^−^ with SO_2_ is also reported for comparative purposes. OIT: oxygen ion transfer; OAT: oxygen atom transfer; DOT: double oxygen transfer.

Reaction	k_dec_ (×10^−10^) ^§^	k/k_coll_ (%)	OIT	OAT	DOT
ClO_2_^−^ + SO_2_ [47]	9.10	63.8	86.5	4.2	9.3
[Cl·Na·ClO_2_]^−^ + SO_2_	2.88	24.2	74.2	8.8	17.0
[F·Na·ClO_2_]^−^ + SO_2_	3.75	30.8	89.2	3.7	7.1
[Br·Na·ClO_2_]^−^ + SO_2_	2.12	18.7	73.4	10.1	16.5
[I·Na·ClO_2_]^−^ + SO_2_	1.85	16.8	68.9	13.7	17.4
[Cl·Li·ClO_2_]^−^ + SO_2_	1.81	14.5	84.7	7.9	7.4
[ClO_2_·Na·ClO_2_]^−^ + SO_2_	7.48	66.0	81.8	4.8	13.4
[SO_3_·Na·ClO_2_]^−^ + SO_2_	3.74	33.0	70.4	6.9	22.7
[(NaClO_2_)_2_·ClO_2_]^−^ + SO_2_	7.39	68.1	-	-	-

^§^ ±30%.

**Table 2:** Rate constants (cm^3^ mol^−1^ s^−1^) for the reactions of the [Cl·Na·ClO_2_]^−^ anion with SO_2_. OIT: oxygen ion transfer; OAT: oxygen atom transfer; DOT: double oxygen transfer.

Reactivity	k × 10^−10^ cm^3^ mol^−1^ s^−1 §^		
OIT	k_1_	k_2.2_	
	2.13	0.743	
OAT	k_2_	k_2.1_	
	0.251	7.53	
DOT	k_3_	k_4_	k_5_
	0.306	0.147	0.034

^§^ ±30%.

**Table 3:** Rate constants (cm^3^ mol^−1^ s^−1^) for the reaction: [ClO_2_·Na·ClO_2_]^−^ + SO_2_. Only the most relevant rate constants are reported. OIT: oxygen ion transfer; OAT: oxygen atom transfer; DOT: double oxygen transfer; add: addition reaction.

Reactivity	k × 10^−10^ cm^3^ mol^−1^ s^−1 §^			
OIT	k_6_			
	6.26			
OAT	k_7_	k_7.1_		
	0.364	9.57		
DOT	k_8_	k_9_	k_10_	k_11_
	0.251	0.417	0.134	0.222
SO_2_ add	k_SO2_			
	0.574			

^§^ ±30%.

**Table 4:** Rate constants (cm^3^ mol^−1^ s^−1^) for the reaction [ClO_2_·Na·SO_3_]**^·^**^−^ + SO_2_. Only the most relevant rate constants are reported. OIT: oxygen ion transfer; OAT: oxygen atom transfer; DOT: double oxygen transfer; add: addition reaction.

Reactivity	k × 10^−10^ cm^3^ mol^−1^ s^−1 §^		
OIT	k_12_		
	2.63		
OAT	k_13_	k_13.1_	
	0.260	1.09	
DOT	k_14_	k_15_	k_16_
	0.167	0.222	0.457
SO_2_ add	k_add_		
	0.494		

^§^ ±30%.

## Data Availability

Not applicable.

## Supplementary Materials

The following are available online. Figure S1: Full-scan mass spectrum of a NaClO_2_ salt solution; Figure S2: Ion-molecule reaction between isolated [Cl·Na·ClO_2_]^−^ cluster ion and SO_2_; Figure S3: CID mass spectrum of [Cl·Na·SO_3_]**^·^**^−^ ion at *m*/*z* 138; Figure S4: CID mass spectrum of [Cl·Na·SO_4_]**^·^**^−^ ion at *m*/*z* 154; Figure S5: CID mass spectrum of [Cl·SO_4_]^−^ ion at *m*/*z* 131; Figure S6: ion-molecule reaction between isolated [ClO_2_·Na·ClO_2_]^−^ cluster ion at *m*/*z* 157 SO_2_; Figure S7: CID mass spectrum of [ClO_2_·Na·SO_3_]**^·^**^−^ product ion at *m*/*z* 170; Figure S8: CID mass spectrum of (a) [ClO·Na·ClO_2_]^−^ product ion at *m*/*z* and (b) [Cl·Na·ClO_3_]^−^ standard ion at *m*/*z* 141; Figure S9: mass spectrum of the ion-molecule reaction of (a) [ClO·Na·ClO_2_]^−^ ion at *m*/*z* and (b) [Cl·Na·ClO_3_]^−^ standard ion at *m*/*z* 141 towards SO_2_; Figure S10: mass spectrum of the ion-molecule reaction between [Cl·Na·ClO_2_]^−^ consecutive product ion at *m*/*z* 125, MS^n^-isolated from the reaction sequence *m*/*z* 157 to *m*/*z* 141 to *m*/*z* 125 and SO_2_; Figure S11: magnified plot of the kinetic reported in Figure 4; Figure S12: mass spectrum of the ion-molecule reaction of (a) [Na·SO_4_]^−^ product ion at *m*/*z* 119, MS^n^-isolated, and (b) [Na·SO_4_]^−^ standard ion at *m*/*z* 119 towards SO_2_; Figure S13: mass spectrum of the ion-molecule reaction between [ClO_2_·Na·SO_3_]^−^ product ion at *m*/*z* 170, MS^n^-isolated from the reaction sequence *m*/*z* 157 to *m*/*z* 170 and SO_2_; Figure S14: CID mass spectrum of product ion at *m*/*z* 183; Figure S15: ion-molecule reaction of (**a**) a mixed ionic population at *m*/*z* 154, MS^n^-isolated from the reaction sequence *m*/*z* 157 to *m*/*z* 170 to *m*/*z* 159 and (b) [Cl·Na·SO4]− ion at *m*/*z* 154, MSn-isolated from the reaction of [Cl·Na·ClO2]− reactant ion towards SO2; Figure S16: mass spectrum of the ion-molecule reaction between [Na·SO_4_]^−^ product ion at *m*/*z* 119 and SO_2_; Scheme S1: Complete reaction sequences of [ClO_2_·Na·ClO_2_]^−^ ion at *m*/*z* 157 and of its product ion [ClO_2_·Na·SO_3_]**^·^**^−^ ion at *m*/*z* 170.

## Sample Availability

Samples are not available from the authors.
