# Supplementary material for: Intracluster Sulphur Dioxide Oxidation by Sodium Chlorite Anions: A Mass Spectrometric Study
Source: Molecules. 2021 Nov 24;26(23):7114. doi: 10.3390/molecules26237114 (PMC8659277; doi:10.3390/molecules26237114)
Supplement: Supplementary file 1 [file molecules-26-07114-s001.zip › molecules-1471488-supplementary.pdf]

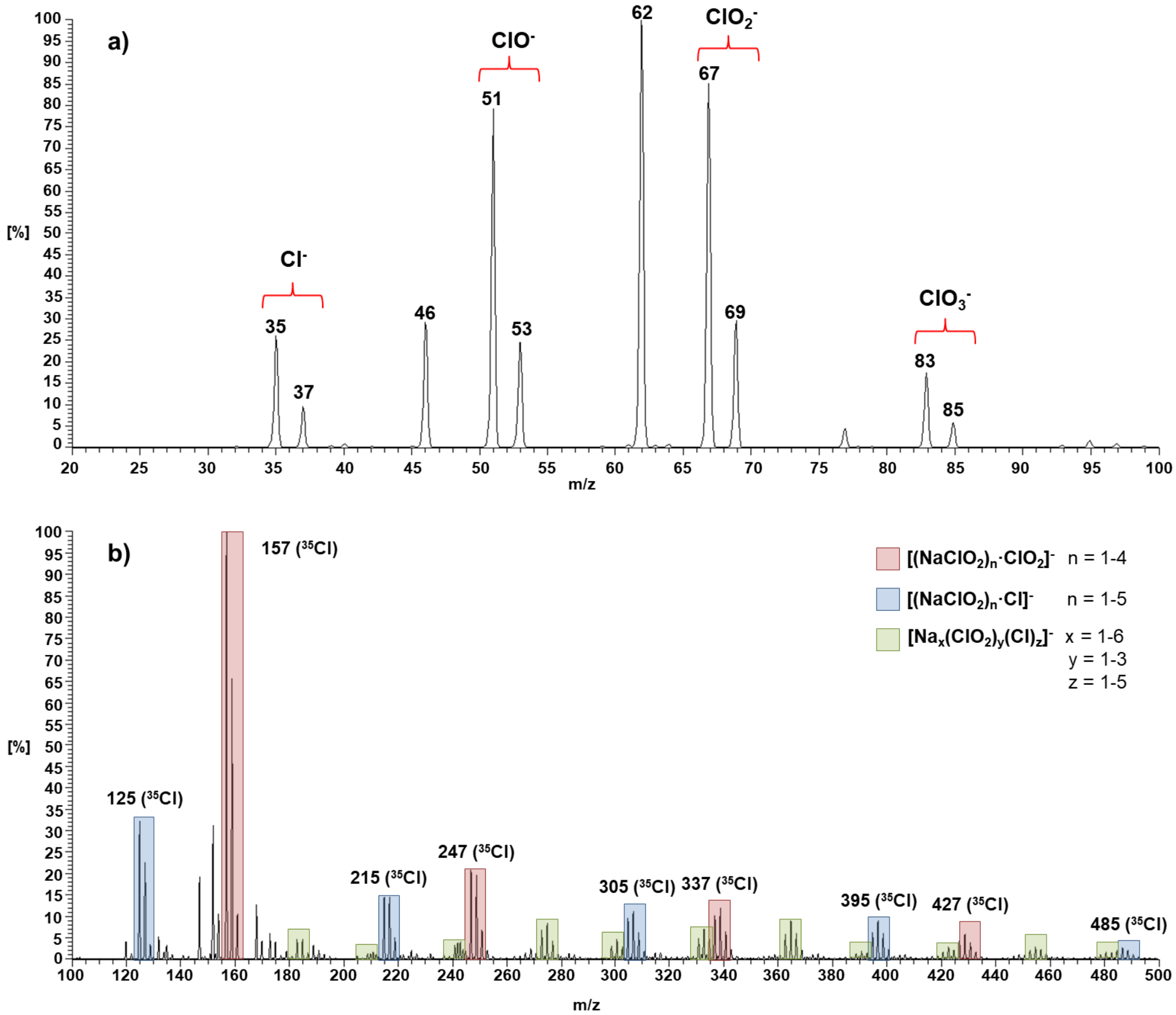

Figure S1. IT full-scan mass spectrum of a millimolar solution containing $NaClO_2$ salt in H_2O/CH_3CN (V/V% 1:1) acquired in the a) 20-100 and b) 100-500 *m/z* range. In spectrum a) ions at *m/z* = 46 and 62 are attributed to NO_2^- and NO_3^- respectively.

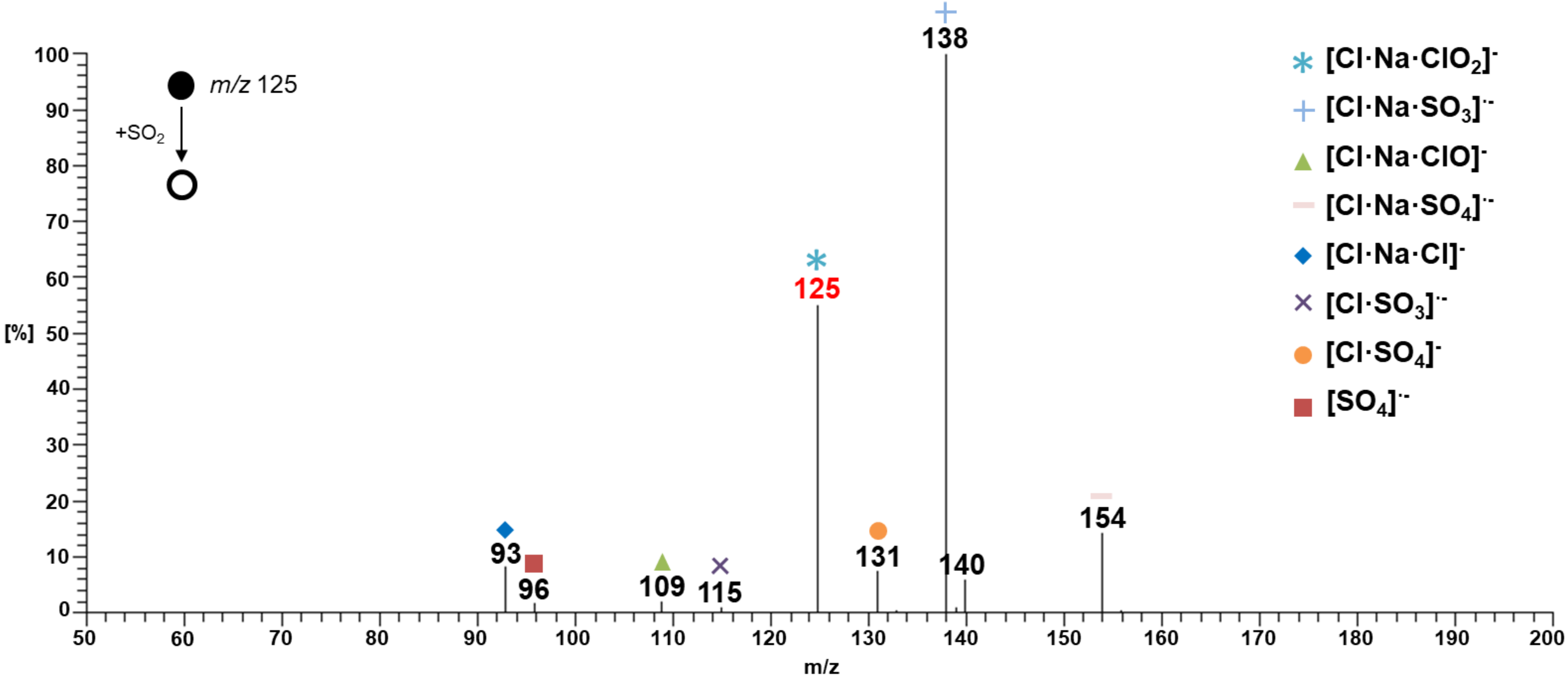

Figure S2. IT mass spectrum of the ion-molecule reaction between isolated $[Cl\cdot Na\cdot ClO_2]^-$ cluster ion at *m/z* 125 (in red) and SO_2 at an activation time (AT) of 700 ms and a P SO_2 = 1.97 x 10^{-7} Torr.

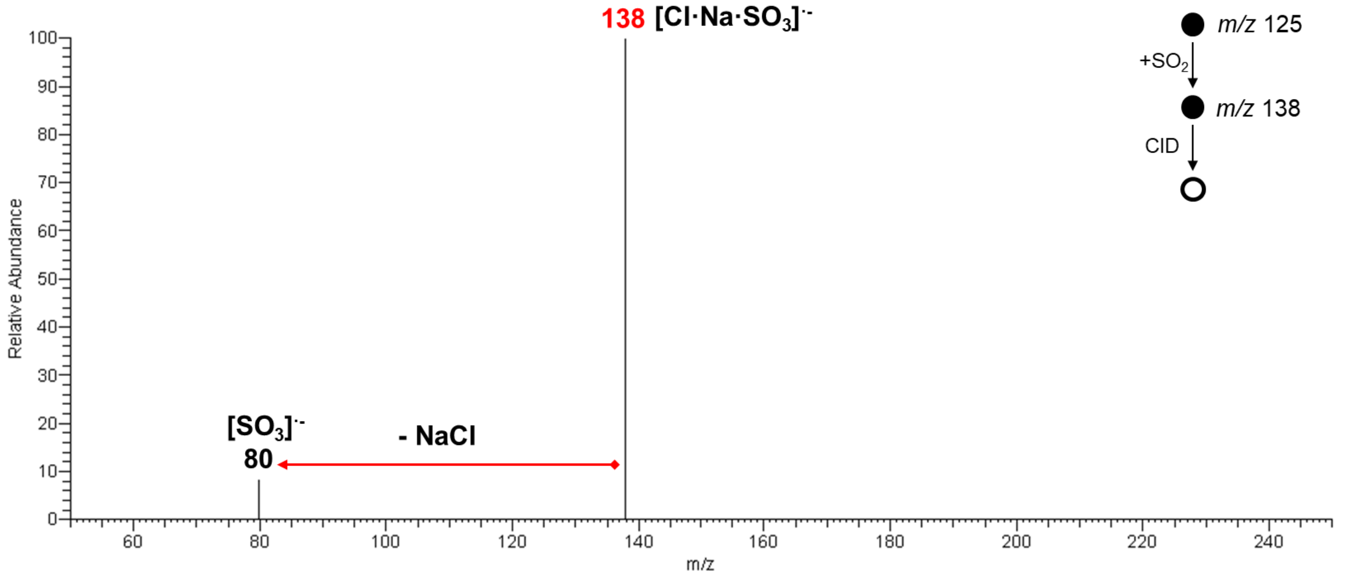

Figure S3. IT-CID mass spectrum of [Cl·Na·SO_3]$^{\cdot-}$ product ion at *m/z* 138 (in red) MS^n -isolated from the reaction of [Cl·Na·ClO_2]$^-$ reactant ion (*m/z* 125) with SO_2.

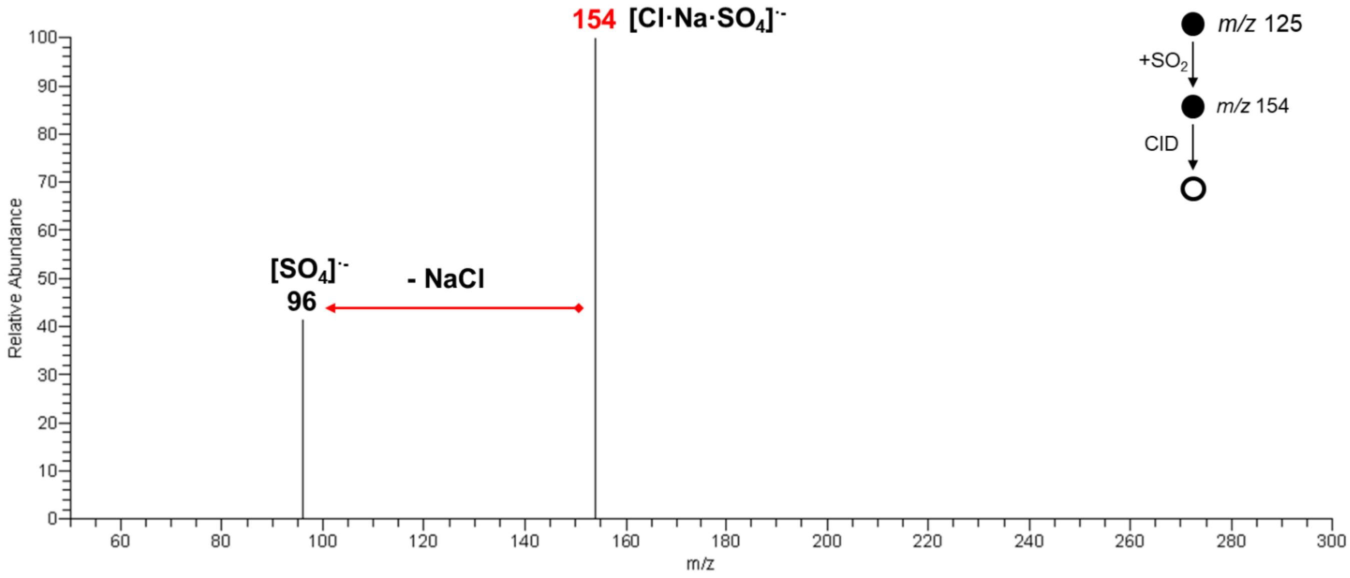

Figure S4. IT-CID mass spectrum of [Cl·Na·SO_4]$^{\cdot-}$ product ion at *m/z* 154 (in red) MS^n -isolated from the reaction of [Cl·Na·ClO_2]$^-$ reactant ion (*m/z* 125) with SO_2.

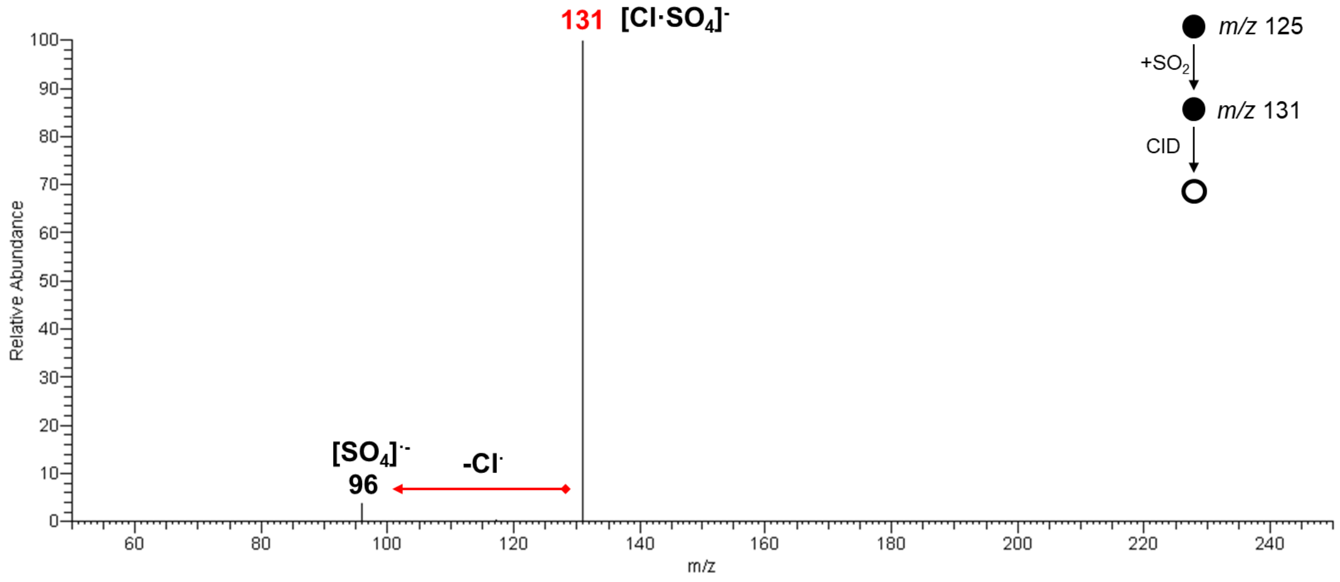

Figure S5. IT-CID mass spectrum of $[Cl{\cdot}SO_4]^-$ product ion at *m/z* 131 (in red) MS^n -isolated from the reaction of $[Cl{\cdot}Na{\cdot}ClO_2]^-$ reactant ion (*m/z* 125) with SO_2.

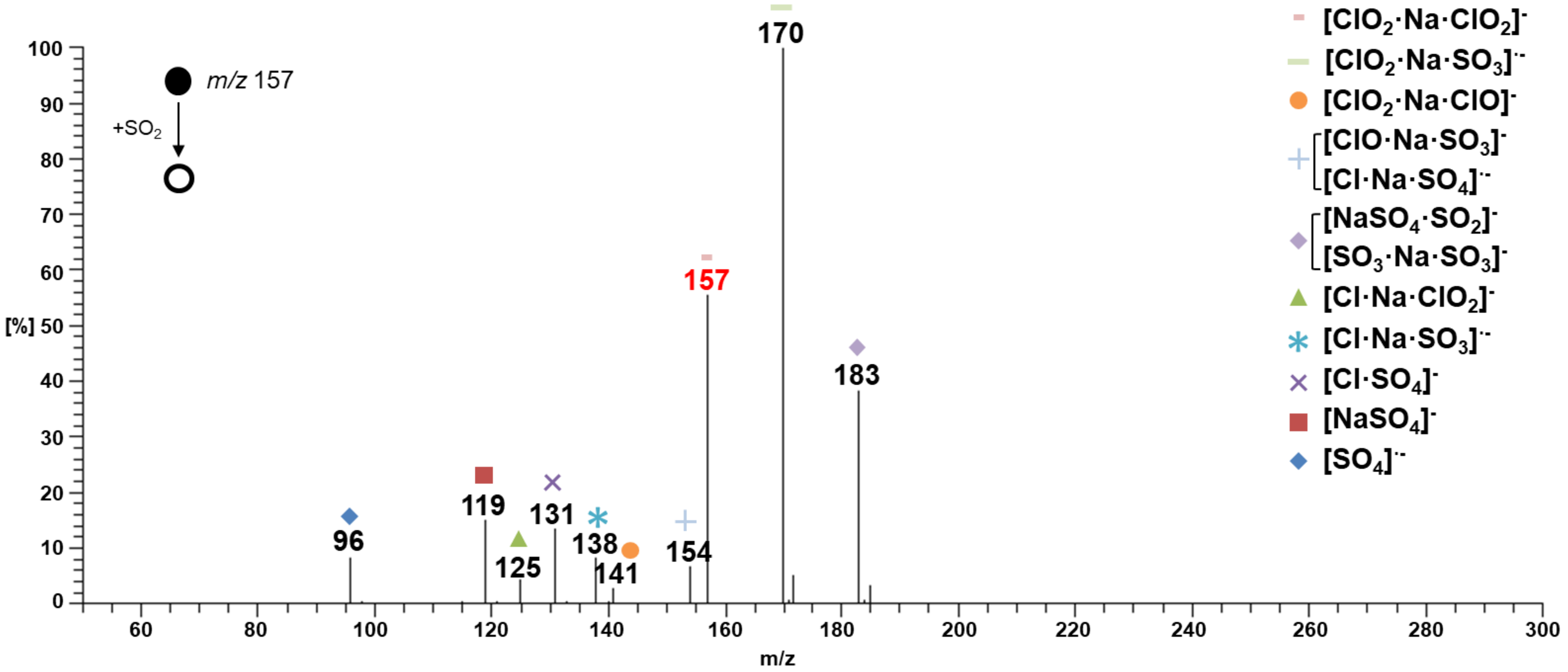

Figure S6. IT mass spectrum of the ion-molecule reaction between isolated $[ClO_2{\cdot}Na{\cdot}ClO_2]^-$ cluster ion at *m/z* 157 (in red) and SO_2 at an activation time (AT) of 350 ms and a P SO_2 = 1.64×10^{-7} Torr.

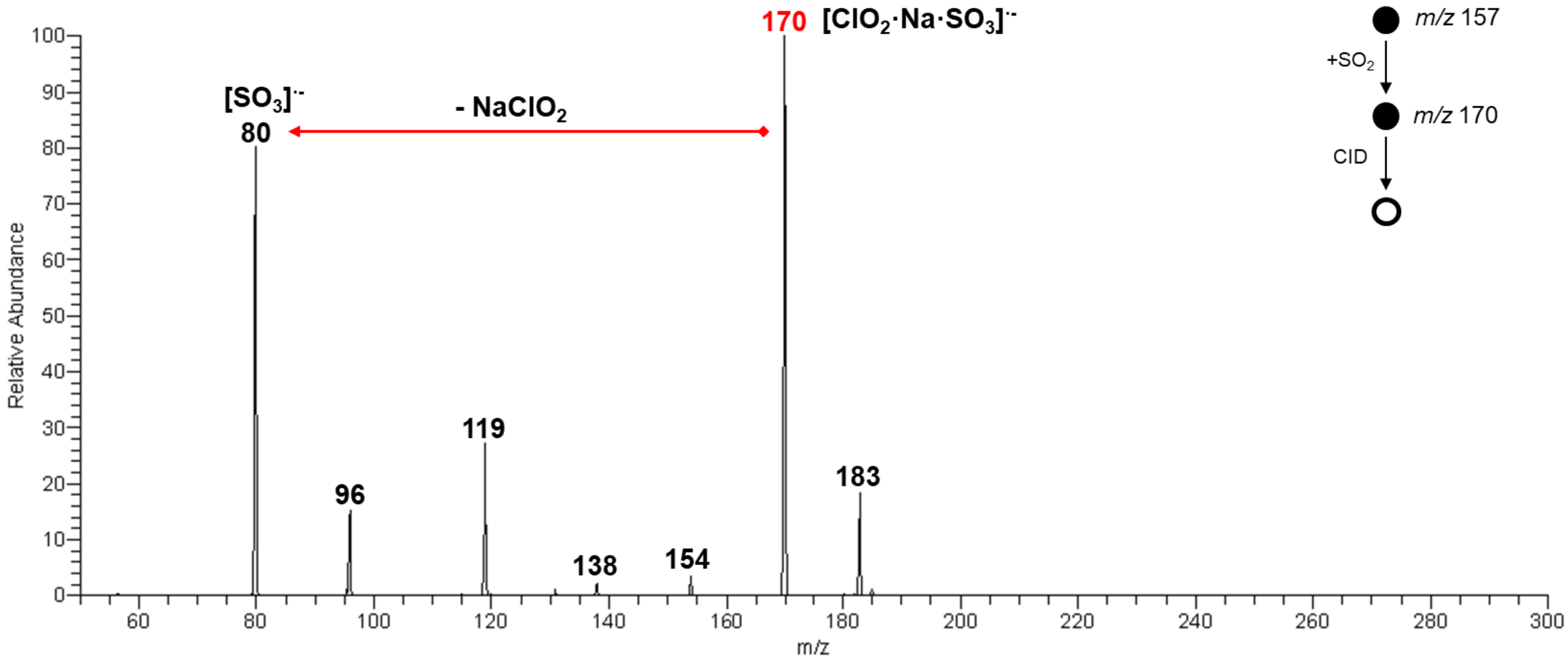

Figure S7. IT-CID mass spectrum of $[ClO_2{\cdot}Na{\cdot}SO_3]^{\cdot-}$ product ion at *m/z* 170 (in red) MS^n -isolated from the reaction of $[ClO_2{\cdot}Na{\cdot}ClO_2]^-$ reactant ion (*m/z* 157) with SO_2. Minor ions at *m/z* 96, 119, 138, 154, and 183 are consistent with the consecutive products of the reaction between $[ClO_2{\cdot}Na{\cdot}SO_3]^{\cdot-}$ ion at *m/z* 170 and SO_2.

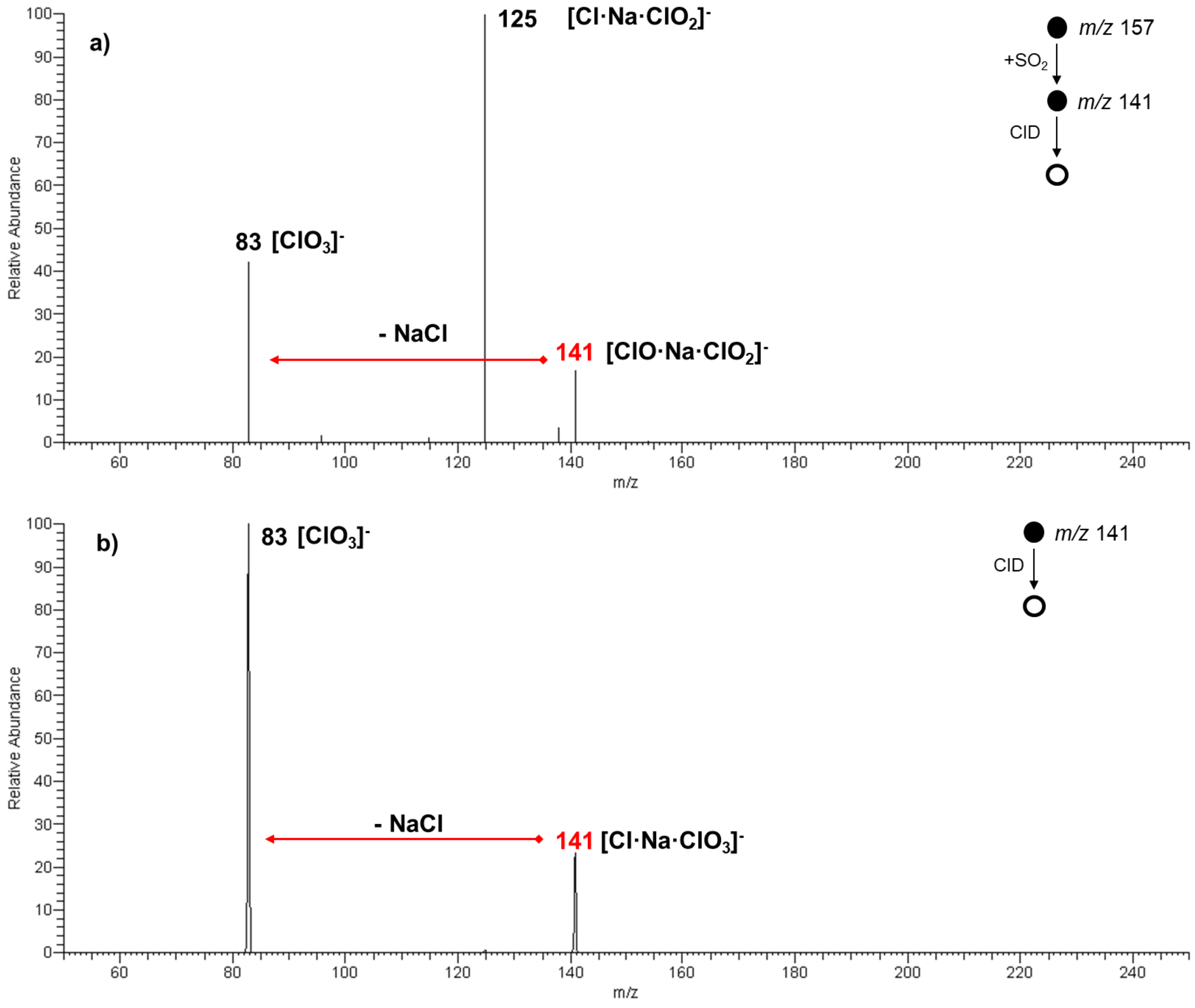

Figure S8. IT-CID mass spectrum of a) $[ClO\cdot Na\cdot ClO_2]^-$ product ion at *m/z* 141 (in red) MS^n -isolated from the reaction of $[ClO_2\cdot Na\cdot ClO_2]^-$ reactant ion (*m/z* 157) with SO_2 and b) $[Cl\cdot Na\cdot ClO_3]^-$ standard ion at *m/z* 141 (in red) obtained from a 1:1 $NaCl/NaClO_3$ millimolar solution.

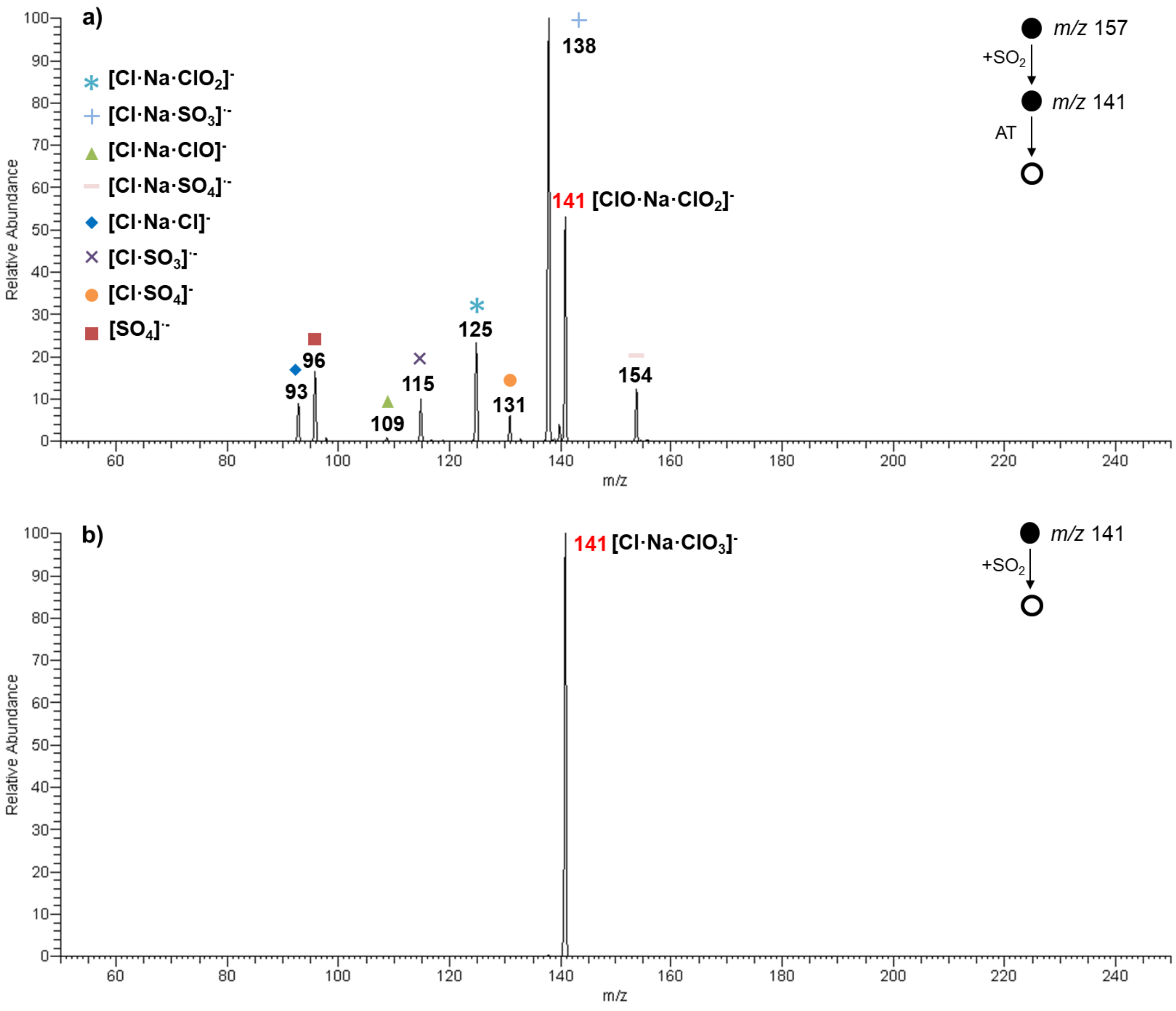

Figure S9. IT mass spectrum of the ion-molecule reaction of a) $[ClO{\cdot}Na{\cdot}ClO_2]^-$ product ion at *m/z* 141 (in red), MS^n -isolated from the reaction of $[ClO_2{\cdot}Na{\cdot}ClO_2]^-$ reactant ion (*m/z* 157) and b) $[Cl{\cdot}Na{\cdot}ClO_3]^-$ standard ion at *m/z* 141 (in red), obtained from a 1:1 $NaCl/NaClO_3$ millimolar solution, towards SO_2 both at an activation time (AT) of 300 ms and a P SO_2 = 5.92 x 10^{-7} Torr.

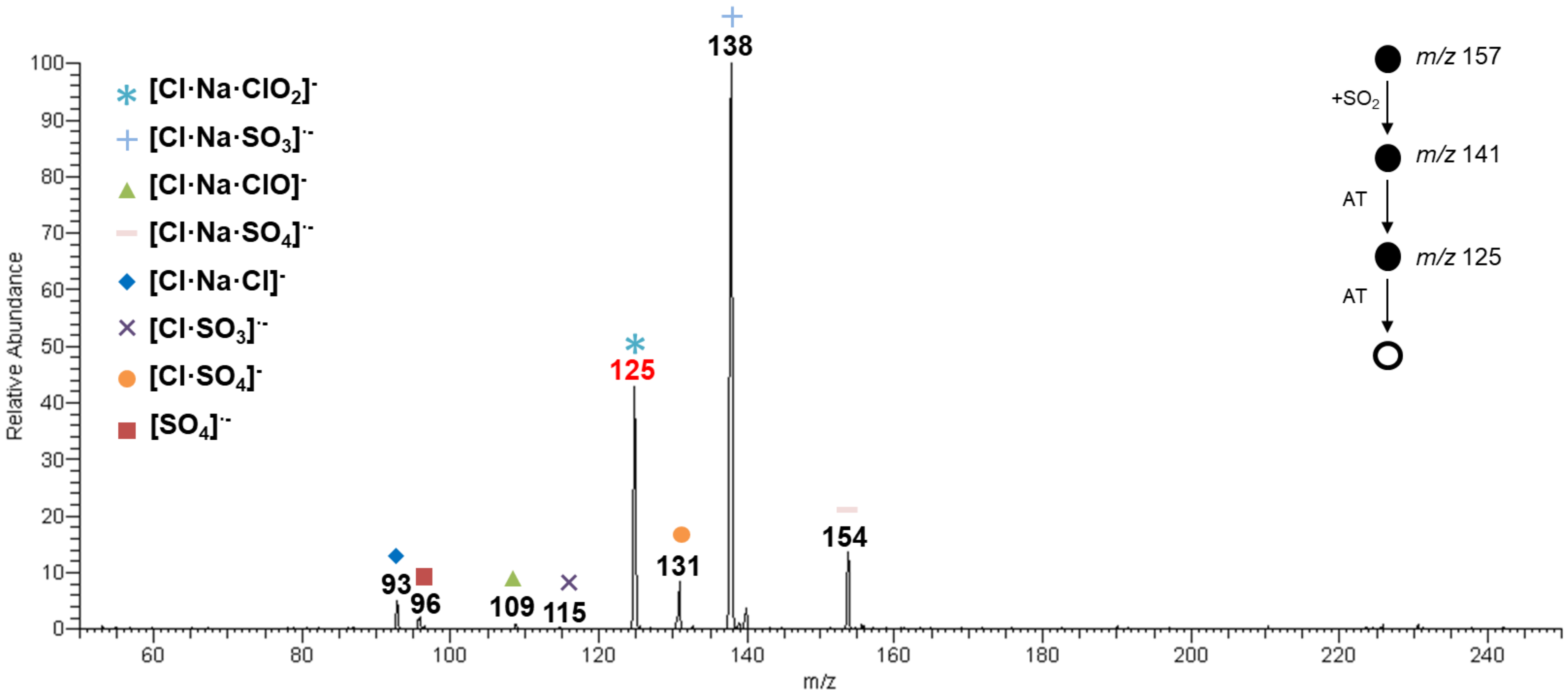

Figure S10. IT mass spectrum of the ion-molecule reaction between $[Cl{\cdot}Na{\cdot}ClO_2]^-$ consecutive product ion at *m/z* 125 (in red), MS^n -isolated from the reaction sequence *m/z* 157 → *m/z* 141 → *m/z* 125 and SO_2 at an activation time (AT) of 200 ms and a P SO_2 = 5.92 x 10^{-7} Torr.

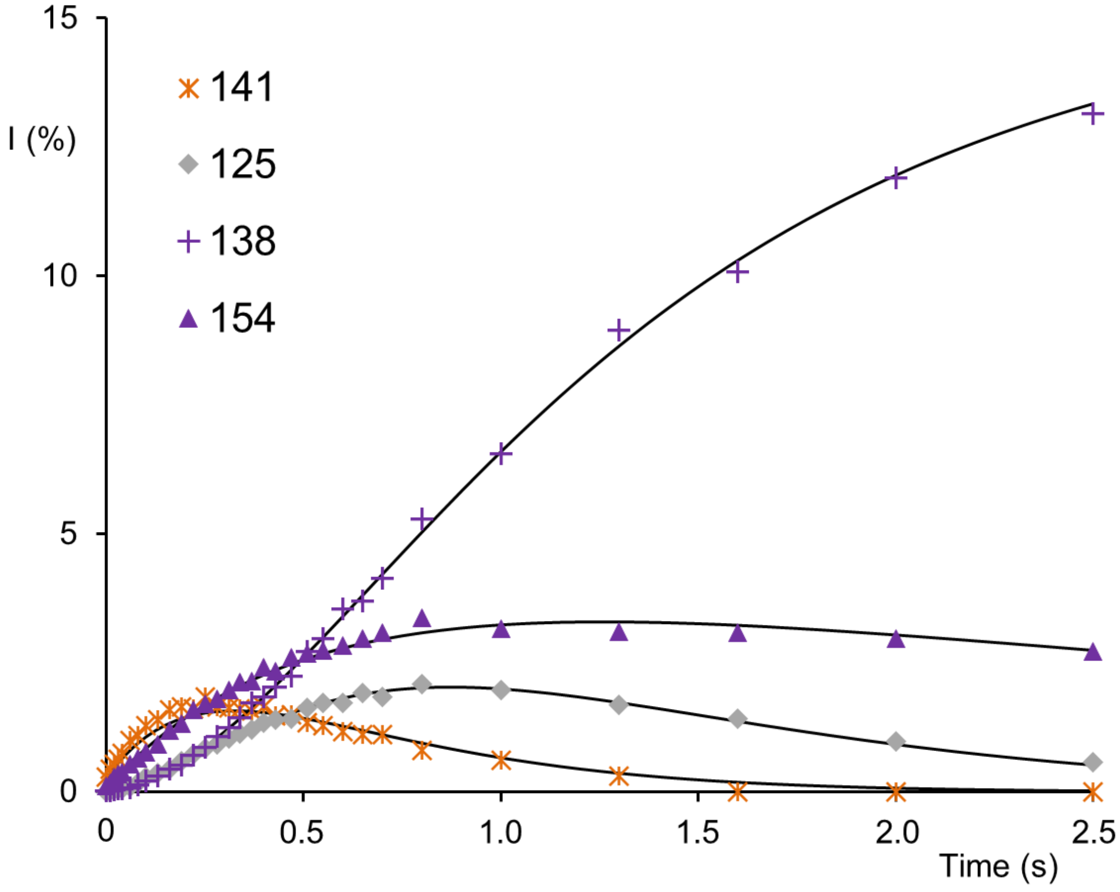

Figure S11. Magnified kinetic plot of the reaction of isolated $[NaClO_2{\cdot}ClO_2]^-$ ions (m/z = 157) with SO_2 taken from Figure 4. Only the time progress of the low signals corresponding to ions at *m/z* = 154, 141, 138 and 125 are shown, related to the sequences: 157 → 170 → 154 → 138 and 141 → 125 → 138.

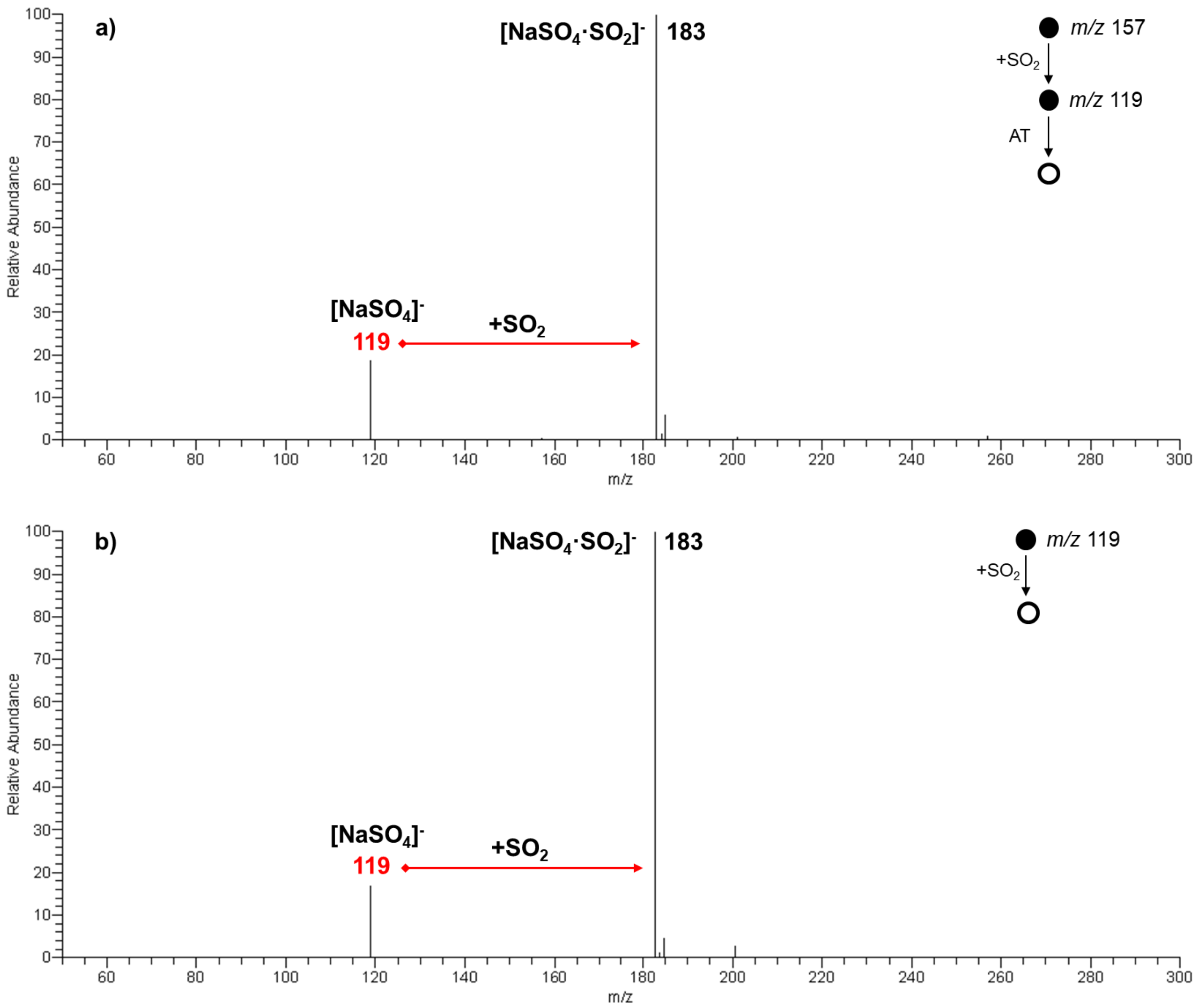

Figure S12. IT mass spectrum of the ion-molecule reaction of a) $[Na{\cdot}SO_4]^-$ product ion at *m/z* 119 (in red), MS^n -isolated from the reaction of $[ClO_2{\cdot}Na{\cdot}ClO_2]^-$ reactant ion (*m/z* 157) with SO_2 (P SO_2 = 2.30 x 10^{-7} Torr); b) $[Na{\cdot}SO_4]^-$ standard ion at *m/z* 119 (in red), obtained from a Na_2SO_4 millimolar solution, towards SO_2 (P SO_2 = 5.20 x 10^{-7} Torr). Both spectra are acquired with at an activation time (AT) of 5000 ms.

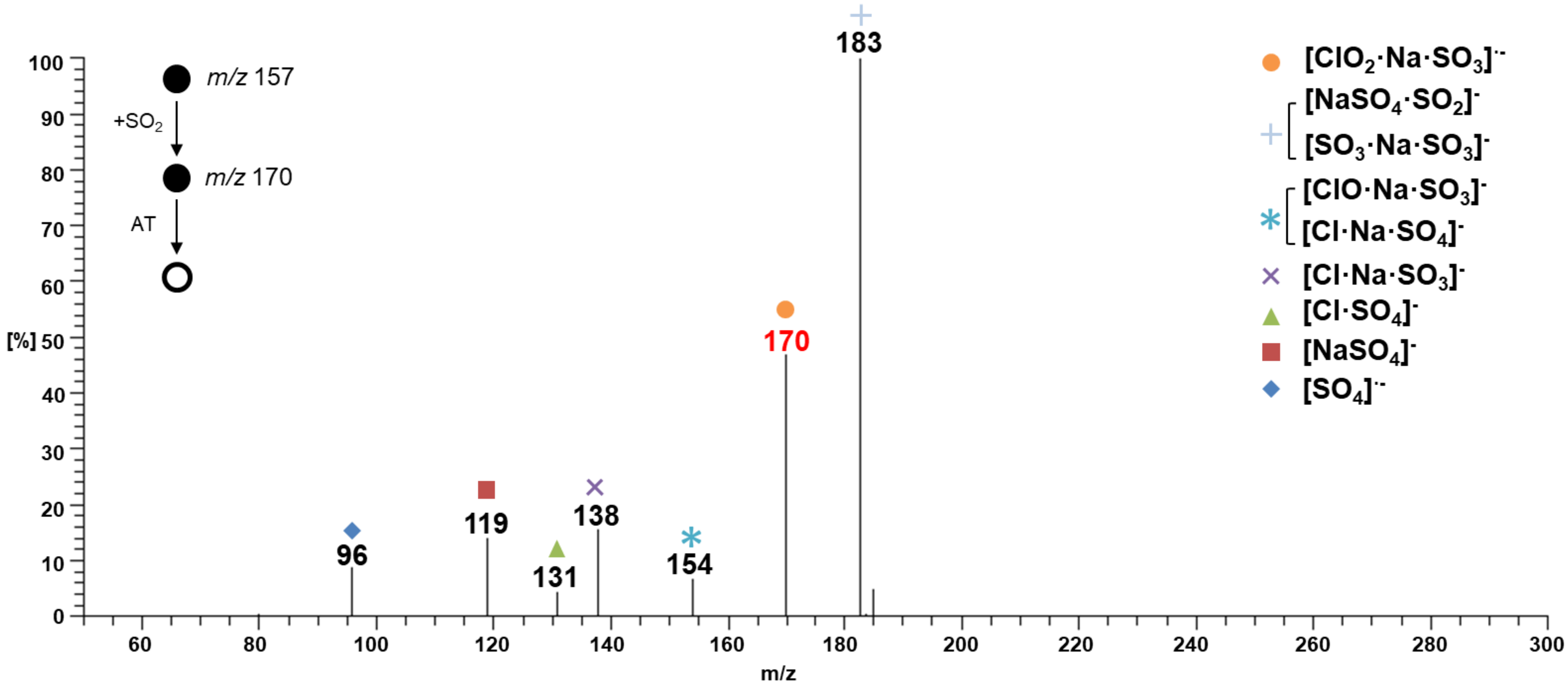

Figure S13. IT mass spectrum of the ion-molecule reaction between $[ClO_2 \cdot Na \cdot SO_3]^{\cdot -}$ product ion at *m/z* 170 (in red) MS^n -isolated from the reaction sequence *m/z* 157 → *m/z* 170 and SO_2 at an activation time (AT) of 700 ms and a P SO_2 = 1.64 x 10^{-7} Torr.

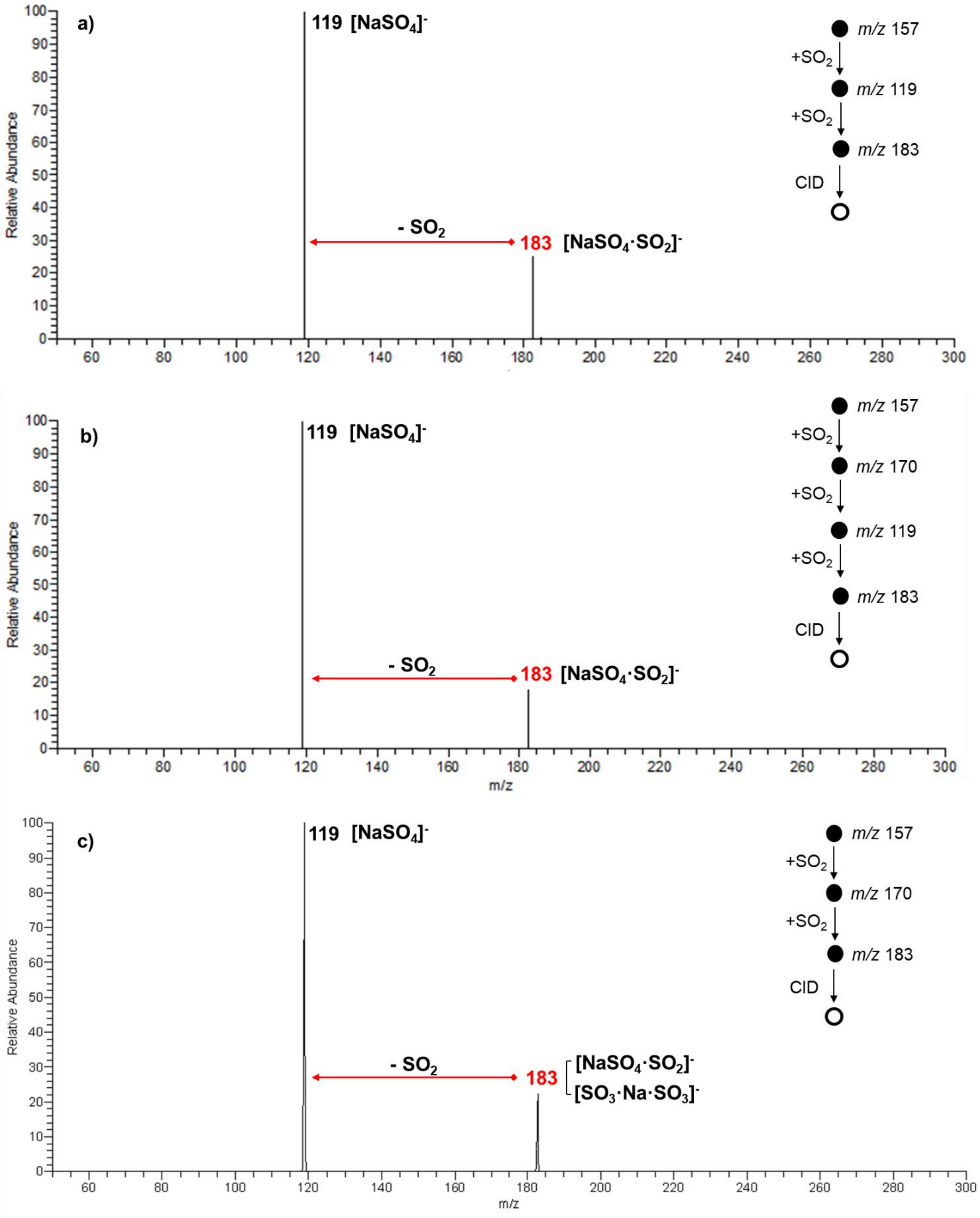

Figure S14. IT-CID mass spectrum of product ion at *m/z* 183 (in red) MS^n -isolated from the reaction sequence a) *m/z* 157 → *m/z* 119 → *m/z* 183, b) *m/z* 157 → *m/z* 170 → *m/z* 119 → *m/z* 183 and *m/z* 157 → *m/z* 170 → *m/z* 183.

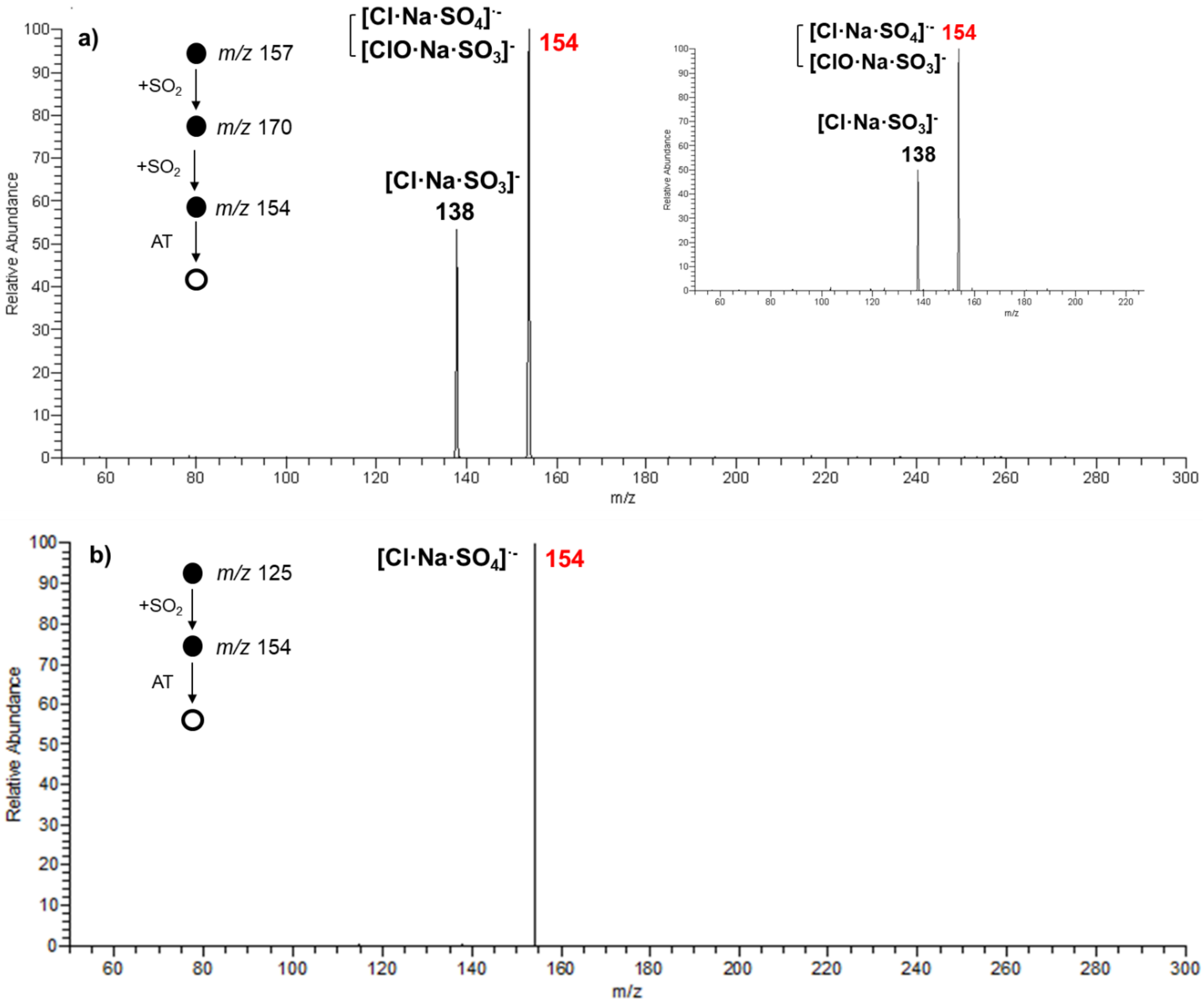

Figure S15. IT mass spectrum of the ion-molecule reaction of a) a mixed ionic population at *m/z* 154 (in red), MS^n -isolated from the reaction sequence *m/z* 157 → *m/z* 170 → *m/z* 159 with SO_2 (P SO_2 = 6.25 x 10^{-7} Torr) b) [Cl·Na·SO_4]$^{·-}$ ion at *m/z* 154 (in red) MS^n -isolated from the reaction of [Cl·Na·ClO_2]$^-$ reactant ion (*m/z* 125), towards SO_2 (P SO_2 = 2.30 x 10^{-7} Torr) both at an activation time (AT) of 150 ms. In the inset the AT is 10.000 ms.

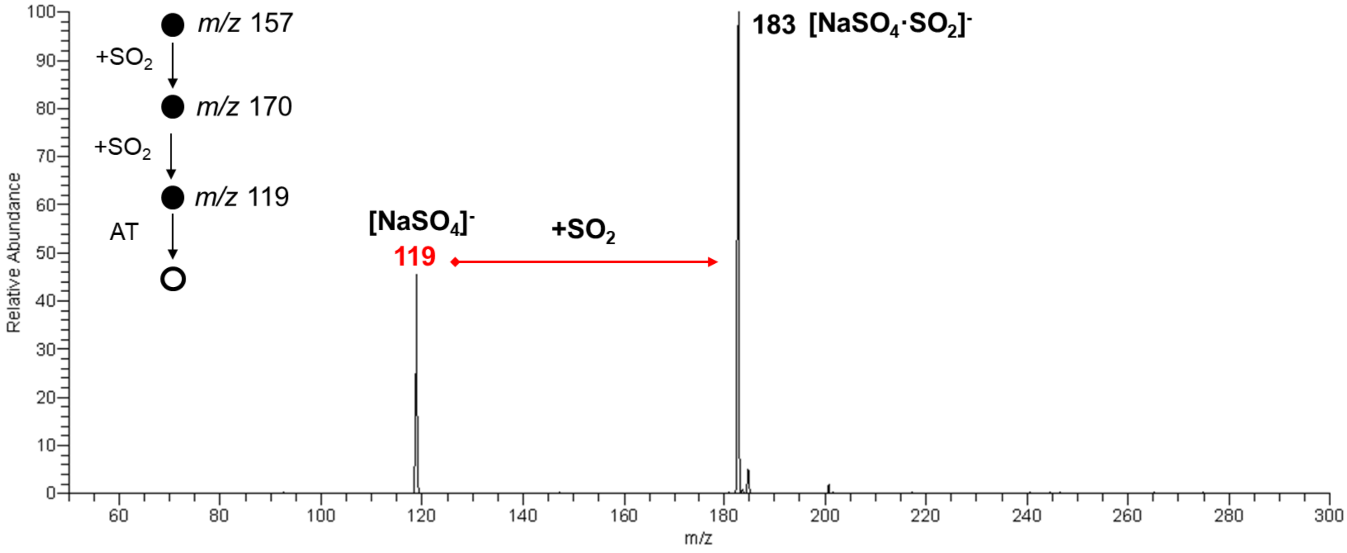

Figure S16. IT mass spectrum of the ion-molecule reaction between $[Na{\cdot}SO_4]^-$ product ion at *m/z* 119 (in red), MS^n -isolated from the reaction sequence *m/z* 157 → *m/z* 170 → *m/z* 119 and SO_2 at an activation time (AT) of 1500 ms and a P SO_2 = 6.25 x 10^{-7} Torr.

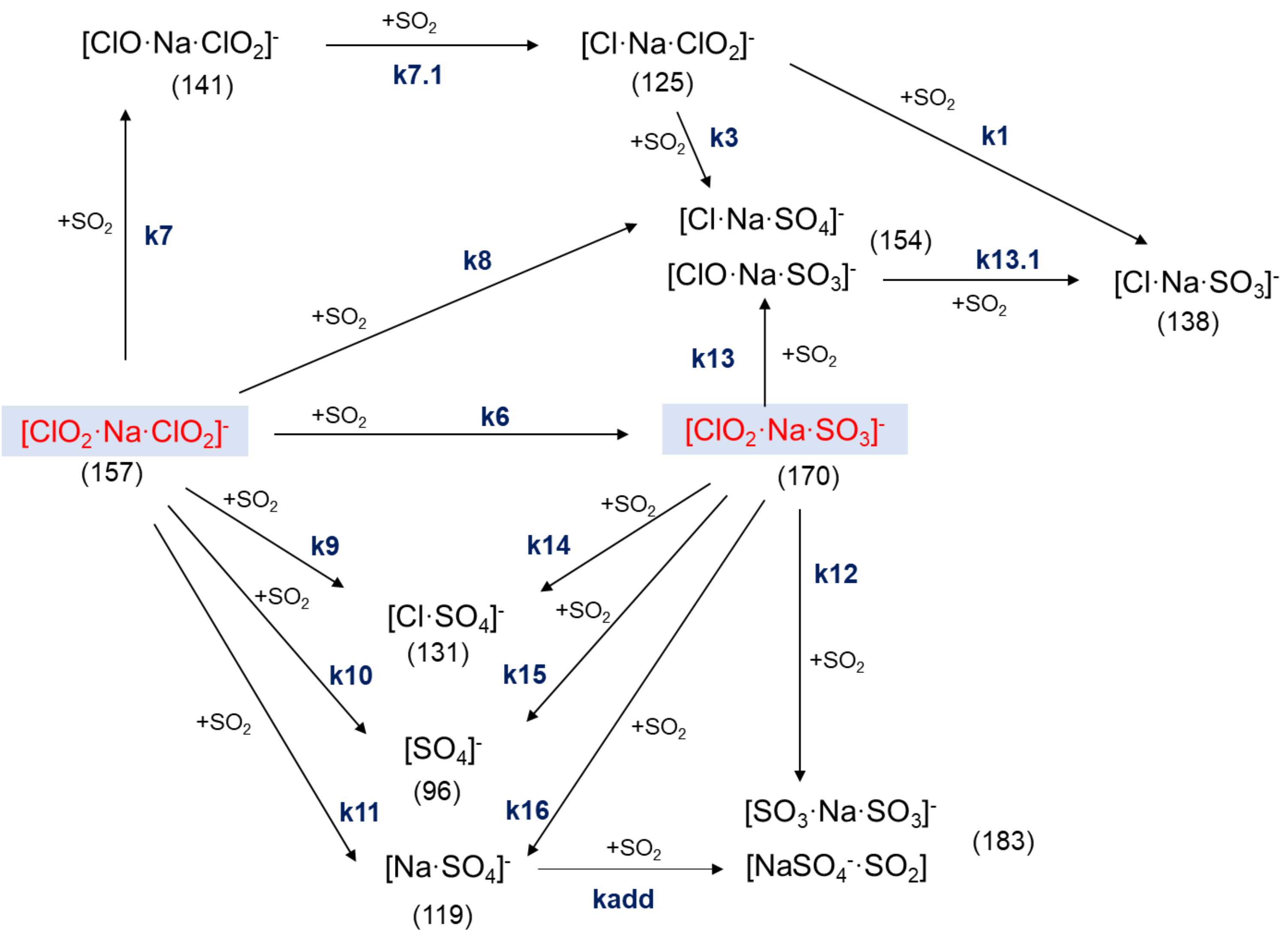

Scheme S1. Complete reaction sequences of $[ClO_2 \cdot Na \cdot ClO_2]^{\cdot -}$ ion at *m/z* 157 and of its product ion $[SO_3 \cdot Na \cdot ClO_2]^{\cdot -}$ ion at *m/z* 170. Rate constants are numbered as in Scheme 1, 2 and 3 (see main text). Losses of the neutral counterparts are omitted for the sake of clarity.
